# SCF + G-CSF treatment in the chronic phase of severe TBI enhances axonal sprouting in the spinal cord and synaptic pruning in the hippocampus

**DOI:** 10.1186/s40478-021-01160-3

**Published:** 2021-04-08

**Authors:** Xuecheng Qiu, Suning Ping, Michele Kyle, Lawrence Chin, Li-Ru Zhao

**Affiliations:** 1 Department of Neurosurgery, The State University of New York Upstate Medical University, 750 E. Adams Street, Syracuse, NY 13210 USA; 2 VA Health Care Upstate New York, Syracuse VA Medical Center, Syracuse, USA

**Keywords:** Traumatic brain injury, Stem cell factor, Granulocyte colony-stimulating factor, Corticospinal tract, Synaptic pruning

## Abstract

Traumatic brain injury (TBI) is a major cause of long-term disability in young adults. An evidence-based treatment for TBI recovery, especially in the chronic phase, is not yet available. Using a severe TBI mouse model, we demonstrate that the neurorestorative efficacy of repeated treatments with stem cell factor (SCF) and granulocyte colony-stimulating factor (G-CSF) (SCF + G-CSF) in the chronic phase is superior to SCF + G-CSF single treatment. SCF + G-CSF treatment initiated at 3 months post-TBI enhances contralesional corticospinal tract sprouting into the denervated side of the cervical spinal cord and re-balances the TBI-induced overgrown synapses in the hippocampus by enhancing microglial function of synaptic pruning. These neurorestorative changes are associated with SCF + G-CSF-improved somatosensory-motor function and spatial learning. In the chronic phase of TBI, severe TBI-caused microglial degeneration in the cortex and hippocampus is ameliorated by SCF + G-CSF treatment. These findings reveal the therapeutic potential and possible mechanism of SCF + G-CSF treatment in brain repair during the chronic phase of severe TBI.

**Supplementary Information:**

The online version contains supplementary material available at 10.1186/s40478-021-01160-3.

## Introduction

Traumatic brain injury (TBI) has become a public health crisis and a major cause of long-term disability and death among children and young adults in the United States [8, 71]. According to severity of the damage to the brain, TBI can be classified into mild, moderate, or severe TBI. Approximately 32 to 40% of TBI cases were classified as severe TBI [13, 24, 51]. The lifetime of medical cost for severe TBI was estimated to be $76.5 billion (in 2010 dollars) in the United States [9]. TBI has been recognized as a chronic medical condition [10, 45] which has long-term even lifelong effects for TBI patients and increases the burden on the TBI survivor’s family and socioeconomic costs [25, 66].

Accumulating evidence shows that TBI-induced progressive neurodegeneration and neural structure disruption persist for several decades [26, 35]. Most permanent impairments have been found in severe TBI and some repeated mild TBI, even a single TBI can cause long-term neurodegeneration [10, 35]. Severe TBI increases the risk of developing depression, dementia, neurodegeneration, and even death during the chronic phase [24, 45]. Current therapeutic strategies for TBI are surgical interventions in the acute phase and rehabilitation post-acute TBI [21, 27]. Due to the improved medical and surgical management, the survival rate following severe TBI has dramatically increased [24]. However, there is still a lack of evidence-based therapy for severe TBI recovery [7, 77], especially in the chronic phase of severe TBI.

Spontaneous neural plasticity events occurring after brain injury, including synaptogenesis, axonal sprouting and neural network remodeling, play an important role in functional recovery [44, 53, 70, 74]. Enhancing adaptive neural plasticity could be a potential therapeutic target for brain repair after TBI. Stem cell factor (SCF) and granulocyte colony-stimulating factor (G-CSF) are two essential hematopoietic growth factors that synergistically regulate the proliferation, differentiation and mobilization of hematopoietic stem/progenitor cells [4, 18, 29]. Increasing evidence has demonstrated the potential efficacy of SCF and G-CSF in brain repair. Our previous studies have shown that combined treatment of SCF and G-CSF (SCF + G-CSF) promotes neurite outgrowth in vitro [22, 69] and enhances neural network remodeling and synaptogenesis in the chronic phase of experimental stroke [11, 12]. SCF + G-CSF treatment in the subacute phase of severe TBI ameliorates TBI-induced neurodegeneration and enhances neural network reorganization [73]. Recently, we have also revealed that SCF + G-CSF treatment in the chronic phase of severe TBI increases neuronal plasticity protein expression, rebalances TBI-induced over-synaptogenesis and enhances neural network remodeling and remyelination [57], indicating the therapeutic potential of SCF + G-CSF on brain repair in the chronic phase of severe TBI.

Using a severe model of TBI in young adult mice, the aim of the present study was to determine whether repeated administration of SCF + G-CSF treatment in the chronic phase of TBI would show a better effect in brain repair than a single treatment. Our findings reveal that SCF + G-CSF-repeated treatments in the chronic phase of severe TBI lead to better neurological function improvement than single SCF + G-CSF treatment. Moreover, SCF + G-CSF treatment promotes corticospinal tract (CST) sprouting, ameliorates severe TBI-induced dendritic spine loss and microglial degeneration, and enhances removal of the severe TBI-induced overgrowth of  synapses by microglial cell-mediated synaptic pruning.

## Materials and methods

All experimental procedures in this study were approved by Institutional Animal Care and Use Committee and performed in accordance with the National Institutes of Health Guide for the Care and Use of Laboratory Animals.

### Mice and treatment

**Fig. 1:**
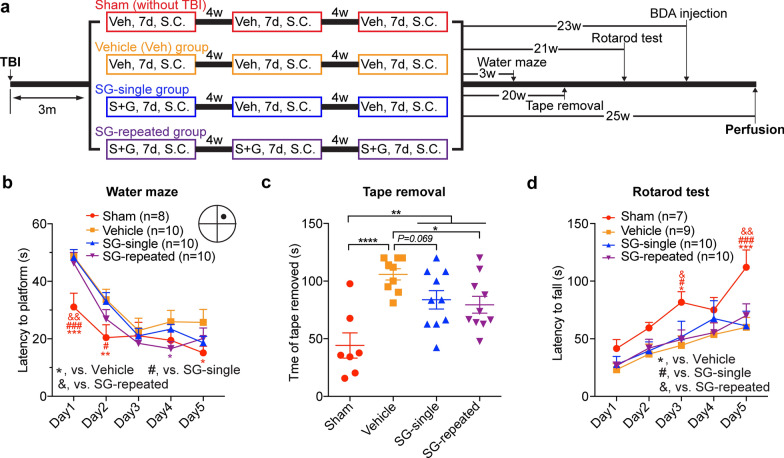
SCF + G-CSF-repeated treatment in the chronic phase of TBI improves neurological function. **a** Experimental flowchart. SC, subcutaneous injection. SG/S + G, SCF + G-CSF treatment. **b** Data of the water maze test. Note that SCF + G-CSF-repeated treatments in the chronic phase of severe TBI significantly improve spatial learning function. The black dot illustrates the location of the platform. Two-way ANOVA followed by Fisher’s LSD test. **c** Data of the adhesive tape removal test. Note that SCF + G-CSF-repeated treatments in the chronic phase of severe TBI significantly improve somatosensory-motor function. One-way ANOVA followed by Fisher’s LSD test. **d** Data of the Rotarod test. Note that there is no improvement after SCF + G-CSF treatment. Two-way ANOVA followed by Fisher’s LSD test. Mean ± SEM. *p < 0.05, ^*#*^*p* < 0.05, ^*&*^*p* < 0.05, **p < 0.01, ^&&^*p* < 0.01, ***p < 0.001, ^*###*^*p* < 0.001, ****p < 0.0001. Sham: n = 7–8, vehicle: n = 9–10, SCF + G-CSF-single treatment: n = 10, SCF + G-CSF-repeated treatment: n = 10

For behavior testing, corticospinal tract tracing and immunohistochemical studies, a total of 40 male C57BL/6 mice (The Jackson Laboratory, Bar Harbor, ME, USA) at the age of 8–10 weeks were randomly divided into four groups: a sham control group, a TBI-vehicle control group, a TBI-SCF + G-CSF-single treatment group, and a TBI-SCF + G-CSF-repeated treatment group (n = 10/group). Twenty-three weeks after the final treatment, half of the mice in each of the four groups (i.e. n = 5/group) were injected with biotinylated dextran amine (BDA) in the contralateral cortex for the TBI mice or in the left cortex for the sham mice to trace the corticospinal tracts. The treatments were initiated 3 months after TBI (i.e. in the chronic phase of TBI). Recombinant mouse SCF (PeproTech, 200 μg/kg/day, dissolved in 0.9% saline) and recombinant human G-CSF (Amgen, 50 μg/kg/day, dissolved in 5% dextrose) were subcutaneously injected for 7 consecutive days. For sham and vehicle control groups, an equal volume of vehicle solution (i.e. 0.9% saline and 5% dextrose) was subcutaneously injected for 7 days. For SCF + G-CSF-repeated treatments, a total of three 7-day- treatment courses with a 4-week interval between each therapeutic course were performed. For SCF + G-CSF-single treatment, a 7-day injection of SCF + G-CSF was subcutaneously performed in the first course of therapy followed by vehicle solution injections in the rest of two courses of therapy. All mice were euthanized at the end of the experiment (i.e. 25 weeks after the final treatment) (Fig. 1a). Due to health concerns, three mice in the sham group and one mouse in the vehicle control group were euthanized before the end of experiment and excluded from this study.

For analysis of the engulfment of synapses by microglia in vivo, an additional nine TBI mice were randomly divided into two groups: a vehicle control group (TBI, n = 4) and an SCF + G-CSF (TBI, n = 5) treatment group. Three months after TBI, mice were given a 7-day treatment as described above. The mice were then sacrificed 24 h after the final injection (Fig. 8a).

All mice were housed under a reversed 12:12 h light–dark cycle (Dark hours: 6:30 am to 6:30 pm. Light hours: 6:30 pm to 6:30am) with ad libitum access to water and a standard laboratory diet.

### Controlled cortical impact model of TBI

A controlled cortical impact model of TBI was performed to make a reproducible and severe TBI as previously described [58]. Briefly, after being anesthetized with Avertin (400 mg/kg, Sigma- Aldrich, St. Louis, MO, USA), mice were immobilized on a stereotaxic instrument (Leica Biosystems Inc., Wetzlar, Germany). A 4-mm-diameter circular craniotomy (centered at 0.0 mm to the bregma and 2.0 mm lateral to the midline) was made on the right side of the skull. The cortex in the right hemisphere was impacted by an electromagnetically driven impactor with a 3-mm diameter flat impact tip (Impact One stereotaxic impactor, Leica Biosystems Inc., Wetzlar, Germany) at a 4° angle to the vertical line with a 1.5 m/s strike speed, 2 mm impact depth from the surface of the dura and 8500 ms dwell time. After surgery, mice were allowed to fully recover on a homeothermic blanket set at 37 °C before transferring to their home cages. Sustained-release buprenorphine (0.6 mg/kg) was subcutaneously injected to alleviate pain after surgery.

### Neurobehavioral tests

Mice were brought to the testing room (using red light for illumination) at least 30 min prior to testing to allow acclimation to the new environment. The behavior tests were started about 8:30 a.m. and ended before 2:00 p.m. The ANY-Maze Video Tracking System (Stoelting Co.) was used for recording mouse performance during the tests.

To test spatial learning, the Morris water maze test was performed 3 weeks after the final repeated SCF + G-CSF treatments as previously described [58]. In brief, mice were tested in a water tank (1.2 m in diameter) filled with water (room temperature) mixed with nontoxic white paint to cover the platform (12 cm in diameter, 1 cm beneath the water) which was set in the center of one of four imaginary quadrants in the tank. Mice were tested for five consecutive days. Each day, mice were examined in four trials (1 min/trail) with a random start from one of the four quadrants. On the first day, mice were held on the platform for 15 s after each trial for training. The average latency to find the platform each day was analyzed.

To evaluate somatosensory-motor deficits, the adhesive tape removal test as described elsewhere [58] was performed. Briefly, on day 1, mice were habituated in a testing beaker for three trials (2 min/trial). On day 2, mice were tested in the same beaker, and two 6-mm circular stickers were pasted onto the palm of each forepaw. The latency to take off each sticker from the forepaws by their mouth was recorded. Each mouse was tested for three trials with a 15 min rest period between trials.

The Rotarod test was used to evaluate motor learning and coordination using a rotarod apparatus (Coulbourn Instrument, Holliston, MA, USA). Mice were placed on the rod at 0 rotation per minute (rpm). The rod was then started at 4 rpm with a linear acceleration (4 rpm every 30 s) until it reached 40 rpm. Each trial was ended when the mouse spun around or fell off the rod. Each mouse was evaluated for five consecutive days. Each day three trials with a 15-min rest break between the trials were performed. The average fall latency of the three trials was analyzed.

### Corticospinal tract tracing

To trace the corticospinal tract from the contralateral somatosensory-motor cortex, BDA (biotinylated, 10,000 MW, Invitrogen, Carlsbad, CA, USA) was injected into the left sensorimotor cortex 2 weeks before mice were euthanized. Mice were anesthetized with Avertin and placed in a stereotaxic instrument. The dura was exposed, and 10% BDA was stereotaxically injected into two sites (0.75 μL per site) using a Micro4 microsyringe pump controller with a 10 μL Hamilton microsyringe (150 nL/min). The coordinates of the two injection sites were 0.75 mm and − 0.75 mm to the bregma, 1.5 mm lateral to the midline and 1.0 mm deep from the dura. After the completion of injection, the needle was kept in place for another 5 min before retracting. The skin was sutured, and mice were kept on a homeothermic blanket set at 37 °C before transferring to the home cage.

### Tissue processing and immunohistochemistry

Mice were transcardially perfused with 0.01 M phosphate buffered saline (PBS) with 10 U/mL heparin, followed by 10% neutral formalin solution (Sigma Aldrich, St. Louis, MO, USA). Brains and spinal cords were removed and post-fixed in 10% neutral formalin solution overnight at 4 °C. The tissues were dehydrated in 30% sucrose solution in PBS and sectioned (coronal sections, in 30 μm thickness) using a cryostat (Leica Biosystems Inc., Wetzlar, Germany). The sections were stored in a − 30 °C freezer with cryo-protectant solution (0.1 M PB with 30% Ethylene glycol and 30% Glycerol) until used.

For free-floating immunofluorescence staining, sections were rinsed with PBS followed by blocking nonspecific binding using 10% normal donkey serum diluted with 1% IgG-free bovine serum albumin (BSA) (Jackson ImmunoResearch Laboratories, West Grove, PA, USA) and 0.3% Triton X-100 (Sigma- Aldrich, St. Louis, MO, USA) in PBS for one hour at room temperature. A Mouse on Mouse Immunodetection Kit (Vector Laboratories, Burlingame, CA, USA) was used for staining with mouse monoclonal antibodies to block endogenous mouse IgG. Subsequently, the sections were incubated with primary antibodies: rabbit anti-myelin basic protein (MBP, 1:500, Abcam, Cambridge, UK), mouse anti-postsynaptic density protein 95 (PSD-95, 1:500, Novus Biologicals, Centennial, CO, USA), rabbit anti-synaptophysin (SYN, 1:600, Sigma-Aldrich, St. Louis, MO, USA), rabbit anti-Gephyrin (1:500, Thermo Fisher Scientific, Waltham, MA USA), sheep anti-Gephyrin (1:600, Novus Biologicals, Centennial, CO, USA), goat anti-Ionized calcium binding adaptor molecule 1 (Iba1, 1:600, Novus Biologicals, Centennial, CO, USA), and rabbit anti-Purinergic Receptor P2Y12 (P2RY12, 1:1000, Brigham and women’s hospital, Boston, MA, USA). In negative control sections, the primary antibodies were omitted. The primary antibodies were diluted in 1% IgG-free BSA and 0.3% Triton X-100 in PBS and incubated overnight at 4 °C with gentle shaking. After rinsing with PBS, the sections were incubated in the corresponding secondary antibodies for two hours in the dark at room temperature. The secondary antibodies were donkey anti-mouse IgG Alexa Fluor 488/594, donkey anti-rabbit IgG Alexa Fluor 488/594, donkey anti-goat IgG Alexa Fluor 647, and donkey anti-sheep IgG Alexa Fluor 488 (all secondary antibodies, 1:500 dilution, Invitrogen, Carlsbad, CA, USA). Finally, the sections were rinsed with PBS and mounted on Superfrost Plus slides (Thermo Fisher Scientific, Waltham, MA USA) with antifade mounting medium containing 4′,6-diamidino-2-phenylindole (DAPI, Vector Laboratories, Burlingame, CA, USA). To visualize BDA-labeled dendrites or axons, Cy3-conjugated avidin (1:300, Jackson ImmunoResearch Laboratories, West Grove, PA, USA) was used during the secondary antibody incubation as described above. In addition, the spinal cord sections were also stained using an ImmunoCruz® ABC Kit (Santa Cruz Biotechnology, Dallas, TX, USA) to detect BDA-labeled axons according to the manufacturer’s instructions.

### Quantitative image analysis

BDA-labeled corticospinal tract at 5–7 segments of the cervical spinal cord were visualized using immunohistochemistry or immunofluorescence staining. To analyze the number of axons crossing the midline of the coronal spinal cord section, three spinal cord sections from each segment of the spinal cord were chosen, and the number of BDA-labeled sprouting axons were manually counted under a 40 × objective lens of the microscope (Zeiss, Oberkochen, Germany). The average number of sprouting axons per segment was statistically analyzed. For Sholl analysis, the spinal cord sections were imaged using the “Tile scan” mode of a Zeiss LSM 780 confocal microscope (Zeiss, Oberkochen, Germany). The sprouted BDA-labeled axons were traced using the plugin “neuronJ” in Fiji (NIH software) followed by Sholl analysis (a plugin in Fiji) with 10 μm step size started from the central canal of the spinal cord [20, 47, 64].

To quantify the different types of dendritic spines, three brain sections from bregma − 0.5 mm to 0.5 mm (avoided the injection sites) were chosen from each mouse. After staining with Cy3-conjugated avidin, the dendrites from layer 2/3 neurons of the contralateral cortex were imaged using the Zeiss LSM 780 confocal microscope with z-stack scanning (0.3 μm interval, 40 × objective lens with a resolution of 1024 × 1024 pixels). After acquiring the z-stack image, maximum intensity projection (a plugin in Fiji) was used to produce a projection image for analysis. The number of different types of dendritic spines was manually counted. At least 10 dendrites were analyzed for each mouse. The types of dendritic spines were distinguished according to the geometric characteristics of the dendritic spines [60].

To quantitate the relative PSD-95, Gephyrin and Iba1 expression, two fields in the stratum radiatum of hippocampal CA1 and cortex (layer 2–5) were randomly selected and imaged under a 40 × objective lens using the z-stacks scanning mode (1-μm interval, total 18 μm). The background of the images was subtracted from all fluorescent channels (rolling ball radius: 50 pixels), split channels and thresholded using Fiji software. Subsequently, the volumes of PSD-95^+^ and Gephyrin^+^ puncta and Iba1^+^ cells were calculated using the “3D RoiManager” plugin in Fiji [54]. The volumes of PSD-95^+^ and Gephyrin^+^ puncta and Iba1^+^ cells were then normalized to the sham group.

To analyze the morphological changes of microglial cells, brain sections were stained with a microglial cell marker, Iba1. Two fields in both the contralateral and ipsilateral stratum radiatum of hippocampal CA1 and cortex (layer 2–5) were imaged with the Zeiss LSM780 confocal microscope under a 40 × objective lens. Z-stack imaging with a 1-μm z-step was performed (total 18 μm). The acquired z-stack images were projected into a single image using the maximum intensity z-projection. The Iba1^+^ cells with an integral cell body were randomly selected from each field and performed threshold process to generate binarized images. The binarized image was filtered by particle size to remove image noise. The filtered images were analyzed using Sholl analysis from the center of the cell soma with a 2-μm step size using Fiji software. To count Iba1 positive cells, the z-projected images were used. The Iba1 positive cells with integrated cell soma and counterstained with DAPI were quantified using Fiji software.

### In vivo engulfment of synapse analysis

Two brain sections from bregma  − 1.46 to − 2.18 mm were chosen for triple immunostaining with PSD-95, Gephyrin, Iba1 or P2RY12. After immunostaining, two fields in the stratum radiatum of hippocampal CA1 per brain section were acquired using the Zeiss LSM780 confocal microscope (40 × objective lens, 1024 × 1024 pixels of the resolution and 1-μm intervals in z-stacks). To analyze the engulfment of synapses, the volumes of Iba1^+^PSD-95^+^ and Iba1^+^Gephyrin^+^ puncta or P2RY12^+^PSD-95^+^ and P2RY12^+^ Gephyrin^+^ puncta were calculated using the plugin “3D RoiManager” in Fiji software. The engulfing index was presented using the following formulas: (Iba1^+^PSD-95^+^ or Iba1^+^Gephyrin^+^ volume)/(Iba1^+^ volume) or (P2RY12^+^PSD-95^+^ or P2RY12^+^Gephyrin^+^ volume)/(P2RY12^+^ volume).

### Synaptosome preparation

Synaptosomes were purified from adult mouse brain according to a well-established method published elsewhere [19]. Briefly, the whole brain without the cerebellum was homogenized in 10% (w/v) homogenizing buffer (0.32 M sucrose, 1 mM EDTA, 5 nM Tris and 0.25 mM DTT, pH 7.4). The homogenate was centrifuged at 1000*g* for 10 min at 4 °C. The supernatant was diluted and layered over top of homogenizing buffer containing 3%, 10%, 15% and 23% (vol/vol) Percoll, respectively. After a 5 min centrifuge at 31,000 g (4 °C), the fraction between the 15% and 23% Percoll gradient solution was collected, diluted to four times in homogenizing buffer and centrifuged at 20,000*g* (4 °C) for 30 min. The pellet was resuspended in isotonic PBS. The protein concentration was quantified using a Pierce BCA Protein Assay Kit (Thermo Fisher Scientific, Waltham, MA, USA) following the manufacturer’s instructions. A Vybrant™ Multicolor Cell-Labeling Kit (Thermo Fisher Scientific, Waltham, MA, USA) was used to label the synaptosomes based on the manufacturer’s instructions. One microliter DiO was added to 200 µL synaptosome suspension and incubated at 37 °C for 1 min, followed by centrifuging at 20,000*g* (4 °C) for 30 min. The DiO-labeled pellet was resuspended in PBS to be used for testing uptake of synaptosomes by microglia in vitro.

### Primary microglial cell culture

Primary microglial cell cultures were prepared from dissecting cerebral cortex of 1-day-old neonatal C57BL/6 mice based on the protocol developed by Saura and coworkers [61]. Briefly, the cortex was dissected and digested using 0.25% trypsin–EDTA at 37 °C for 30 min. Digested tissue was resuspended in DMEM/F12 with 10% fetal bovine serum (FBS), pipetted into single cells and filtered through a 70-μm cell strainer (Thermo Fisher Scientific, Waltham, MA, USA). The filtered cell suspension was centrifuged at 300*g* for 10 min. Pellets were resuspended in DMEM/F12 with 10% FBS and plated on Poly-d-Lysine (PDL, 100 μg/mL in sterile distilled water, Sigma- Aldrich, St. Louis, MO, USA) coated T75 cell culture flasks. The culture medium was replaced every 4 days until achieving confluency. The mixed glial cultures were then incubated in 0.05% trypsin–EDTA (0.25% trypsin–EDTA diluted in DMEM/F12) for 30–60 min. The detached cells were discarded, and the undetached cells were collected for further study.

### In vitro engulfment of synaptosome assay

For the flow cytometry assay, isolated primary microglial cells were grown on a PDL-coated 24-well plate overnight and then treated with or without SCF + G-CSF (20 ng/mL each) for 48 h. The DiO-labeled synaptosomes were added to each well of cultured microglial cells (final concentration: 1 μg/mL). After a 6-h incubation with synaptosomes, cells were washed with warm PBS (37 °C) three times and detached with 0.25% trypsin–EDTA. After centrifugation, cells were resuspended in ice-cold flow cytometry buffer (2% FBS, 2 mM EDTA in PBS) and directly went through a BD LSRFortessa™ cell analyzer. Data were analyzed using FlowJo software. The DiO positive (DiO^+^) cells and median fluorescence intensity (MFI) were analyzed.

For the immunocytochemistry assay, microglia cells were grown on 12-mm diameter coverslips coated with PDL. The cell concentration was 50,000 cells per coverslip. Cells were then treated with or without SCF + G-CSF (20 ng/mL each) for 48 h. After incubating with DiO-labeled synaptosomes (1 μg/mL) for 6 h, cells were washed with warm PBS (37 °C) and fixed using 10% neutral formalin solution for 10 min (Sigma-Aldrich, St. Louis, MO, USA). After rinsing with PBS, cells were incubated with PE-conjugated CX3CR1 antibody (1:100, BioLegend, San Diego, CA, USA) for 2 h and then washed with PBS three times. Considering Triton-X100 could reduce fluorescence of DiO-labeled synaptosome membrane, the cell membrane receptor marker CX3CR1 was used instead of Iba1 to label microglia.

For the western blot assay, microglial cells were grown on PDL coated six-well plates. Cells were treated with or without SCF + G-CSF (20 ng/mL each) for 48 h. Microglial cells were then incubated with unlabeled synaptosomes (1 μg/mL) for 6 h, rinsed with PBS and lysed in ice-cold lysis buffer (20 mM sodium phosphate, 150 mM sodium chloride, 50 mM sodium fluoride, 5 mM EDTA and 1% Triton X-100 with proteinase inhibitor cocktail) for 30 min. The lysates were centrifuged at 12,000 rpm for 15 min at 4 °C, and the supernatant was collected. The protein concentration was then quantified using the Pierce BCA Protein Assay Kit (Thermo Fisher Scientific, Waltham, MA, USA). The quantified samples were boiled in the loading buffer, electrophoresed in 10% SDS-PAGE gel, and transferred to a nitrocellulose membrane (0.45 μm pore size, Amersham Biosciences GE, Little Chalfont, UK). Protein blots were blocked with 5% non-fat milk for 1 h (room temperature, RT) and probed overnight at 4 °C with rabbit anti-beta-Actin (β-Actin, 1:5000, Sigma-Aldrich, St. Louis, MO, USA), mouse anti-PSD-95 (1:1000, Sigma-Aldrich, St. Louis, MO, USA), and rabbit anti-SYN (1:1000, Sigma-Aldrich, St. Louis, MO, USA) in 5% IgG-free BSA (Jackson ImmunoResearch Laboratories, West Grove, PA, USA) diluted with Tris-buffered saline (TBS). After rinsing with TBS containing Tween-20 (TBS-T, 0.5% Tween-20) three times, the blots were incubated with the corresponding alkaline phosphatase-conjugated goat anti-mouse IgG or goat anti-rabbit IgG (1:10,000, Sigma-Aldrich, St. Louis, MO, USA) for 2 h (RT). The membranes were then washed with TBS-T, incubated with ECF substrate (Sigma-Aldrich, St. Louis, MO, USA) and visualized using the ChemiDoc imaging system (Bio-Rad, Hercules, CA, USA). Proteins were extracted from microglial cells in four independent experiments. The levels of protein expression were quantified using Fiji (ImageJ, NIH software).

### Statistical analysis

Data are presented as mean ± standard error of the mean (SEM). All data were analyzed using GraphPad Prism (GraphPad Software, La Jolla, CA, USA). The data of water maze and rotarod tests were examined by repeated two-way analysis of variance (ANOVA) followed by Fisher’s LSD tests. Sholl analysis data were examined with two-way analysis of variance (ANOVA) followed by Tukey’s post hoc tests. Other data were analyzed using one-way ANOVA followed by Fisher’s LSD tests for multiple group comparisons and Student’s t test for two group comparisons. The Kolmogorov–Smirnov test was used for the comparison of cumulative frequency. Two-tailed statistical significance tests were used throughout, and *p* < 0.05 was considered statistically significant.

## Results

### SCF + G-CSF-repeated treatments in the chronic phase of severe TBI improve neurological function

To determine the neurorestorative efficacy of SCF + G-CSF treatment in the chronic phase of severe TBI, spatial learning and memory were evaluated in a water maze test 3 weeks (i.e. at 26 weeks post-TBI) after the final repeated treatments (Fig. 1a). TBI-vehicle control mice spent significantly longer time to find the hidden platform at day 1 (Fig. 1b, *p* < 0.001), day 2 (Fig. 1b, *p*< 0.01) and day 5 (Fig. 1b, *p* < 0.05) as compared with the sham control mice, indicating that a single severe TBI causes long-term deficits in spatial learning and memory. TBI mice in both the SCF + G-CSF-single and SCF + G-CSF-repeated treatment groups took more time to find the hidden platform at day 1 as compared with the sham group (Fig. 1b, SCF + G-CSF-single vs. sham, *p* < 0.001; SCF + G-CSF-repeated vs. sham, *p* < 0.01). At day 2, TBI mice with SCF + G-CSF-single treatment, but not the TBI mice with SCF + G-CSF-repeated treatments, still showed a significant difference from the sham controls (Fig. 1b, *p* < 0.05). Moreover, TBI mice that received SCF + G-CSF-repeated treatments showed significant reductions in the latency to find the hidden platform as compared with TBI-vehicle controls on day 4 testing (Fig. 1b, *p* < 0.05). These findings suggest that SCF + G-CSF-repeated treatments in the chronic phase of severe TBI improve TBI-induced impairment of spatial learning and memory.

The tape removal test was used to evaluate somatosensory-motor deficits 20 weeks after the final treatment (i.e. 43 weeks after TBI). The latency of tape removal from the affected left forepaw was significantly increased in the TBI-vehicle mice as compared with the sham controls, suggesting that severe TBI induces a persistent impairment in somatosensory-motor function (Fig. 1c, *p* < 0.0001). SCF + G-CSF-single treatment slightly reduced the tape removal latency as compared with the TBI-vehicle mice (Fig. 1c, *p* = 0.069), while SCF + G-CSF-repeated treatments significantly decreased the tape removal latency in comparison with the TBI-vehicle controls (Fig. 1c , *p* < 0.05). The tape removal latency in the TBI mice that received SCF + G-CSF-single and SCF + G-CSF-repeated treatments was still significantly longer than that in the sham group (Fig. 1c, *p* < 0.01). There was no significant difference between SCF + G-CSF-single treatment and SCF + G-CSF-repeated treatment groups (Fig. 1c, *p* > 0.05). These data suggest that SCF + G-CSF treatment in the chronic phase of severe TBI improves the TBI-induced somatosensory-motor function impairment. SCF + G-CSF-repeated treatments show better improvements than the SCF + G-CSF-single treatment.

We also performed the Rotarod test 21 weeks after the final repeated treatment. The length of time that mice stayed on the rotating rod was significantly decreased in all TBI mice treated with or without SCF + G-CSF as compared with the sham controls (Fig. 1d, day 3: *p* < 0.05; day 5: sham vs. vehicle, *p* < 0.001; sham vs. SCF + G-CSF-single, *p* < 0.001; sham vs. SCF + G-CSF-repeated, *p* < 0.01), suggesting that severe TBI induces long-term impairments of motor learning and coordination function. There were no significant differences among the TBI groups (Fig. 1d, *p* > 0.05), indicating that SCF + G-CSF has no effect on motor learning and coordination function recovery. This observation is consistent with our previous studies in the severe TBI model [57, 73], indicating that severe TBI-induced extensive damage in the ipsilateral motor cortex leads to severe and persistent impairments in motor learning and coordination which is difficult to be repaired by SCF + G-CSF treatment.

### SCF + G-CSF treatment in the chronic phase of severe TBI enhances corticospinal tract sprouting

**Fig. 2:**
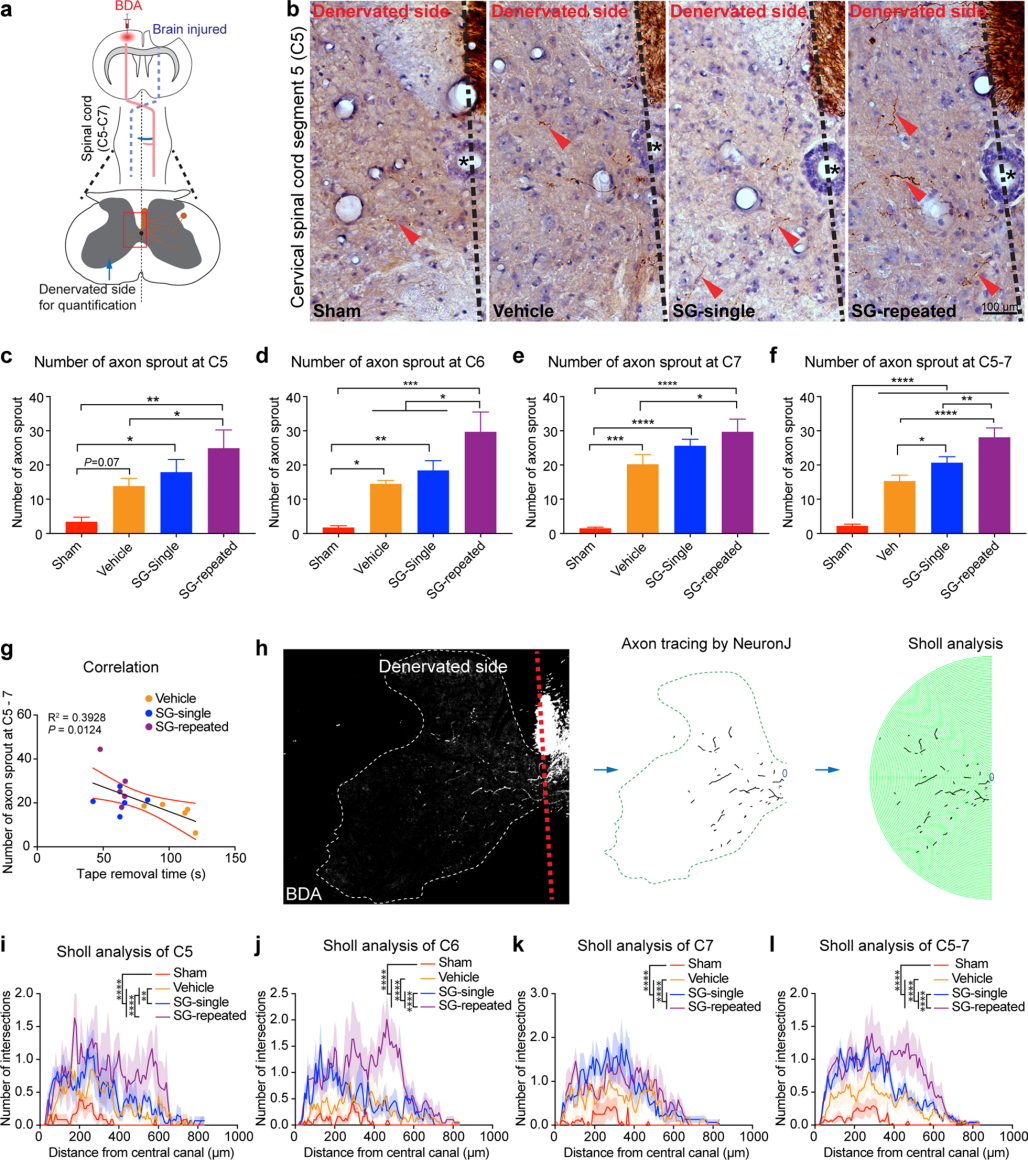
SCF + G-CSF treatment, especially the repeated treatments, in the chronic phase of severe TBI promotes the intact corticospinal tract sprouting in the cervical spinal cord. **a** Schematic illustration of tracking the intact corticospinal tract (CST) at the C5-7 segments of the cervical spinal cord by injection of BDA in the contralesional cortex. The BDA-labeled CST sprouting into the denervated side of cervical spinal cord. The red box in the spinal cord shows the location of representative images in panel **b**. **b** Representative images of immunohistochemistry staining show the intact corticospinal tract sprouting into the denervated side of the cervical spinal cord. The red arrowheads indicate the sprouted corticospinal tract axons (BDA positive brown fibers) in the denervated side of cervical spinal cord. **c**–**f** Quantification of sprouted intact corticospinal tract axons in the denervated side of the C5 (**c**), C6 (**d**), C7 (**e**) and C5-7 segments (**f**). Note that SCF + G-CSF treatment, especially the repeated treatment, significantly enhances the intact corticospinal tract sprouting. One-way ANOVA followed by Fisher’s LSD test. **g** Data of the correlation assay by Pearson’s correlation test. Note that the number of the sprouted intact corticospinal tract axons in the denervated side of C5-7 segments is negatively correlated with the latency of removing the adhesive tape from the affected (left) forepaw. **h** The illustrations show the process of the Sholl analysis for the sprouted corticospinal tract axons in the denervated side of the cervical spinal cord. The sprouted BDA-labeled CST axons were traced using the plugin “neuronJ” in Fiji software followed by Sholl analysis. **i**–**l** Sholl analysis data reveal that SCF + G-CSF treatment, especially the repeated treatments, significantly promotes the intact corticospinal tract sprouting in the C5 (**i**), C6 (**j**), C7 (**k**) and C5-7 (**l**). Two-way ANOVA followed by Tukey’s post hoc test. Mean ± SEM. *p < 0.05, **p < 0.01, ***p < 0.001, ****p < 0.0001. Sham: n = 4, TBI-vehicle: n = 5, TBI-SCF + G-CSF-single treatment: n = 5, TBI-SCF + G-CSF-repeated treatment: n = 5

As stated earlier, our findings from the tape removal test demonstrated the improvement of somatosensory-motor function after SCF + G-CSF treatment. It has been reported that corticospinal tract sprouting contributes to function recovery after brain injury [85]. Therefore, we sought to determine whether SCF + G-CSF treatment enhances corticospinal tract sprouting. The TBI model used in the present study causes severe damage in the ipsilateral (right) somatosensory-motor  cortex, leading to the loss of the corresponding corticospinal tract. To track uninjured corticospinal tract axons, we injected an anterograde neuronal tracer, biotinylated dextran amine (BDA), into the contralesional (left) somatosensory-motor cortex 23 weeks after the final SCF + G-CSF-repeated treatment (i.e. 2 weeks before sacrificing mice) (Figs. 1a, 2a). In the sham mice, after crossing in the pyramidal decussation most BDA-labeled axons appeared to the right side of the cervical spinal cord; only a few axons appeared on the left side (Fig. 2b, Additional file 1: Fig. S1 and Additional file 2: Fig. S2). By contrast, in the TBI-vehicle control mice, substantial numbers of BDA-labeled corticospinal tract axons crossed the midline of the cervical spinal cord and extended into the denervated side (left side) of the cervical spinal cord as compared with the sham controls (Fig. 2b–f, sham vs. vehicle: C5, *p* = 0.07; C6, *p* < 0.05; C7, *p* < 0.001; C5-7, *p* < 0.0001). Although there were no significant differences between TBI-SCF + G-CSF-single treatment and TBI-vehicle controls in the C5, C6 and C7 separately, SCF + G-CSF-single treatment significantly increased corticospinal tract sprouting as compared with the TBI-vehicle controls when we combined all data from C5, C6 and C7 together (Fig. 2f, *p* < 0.05). Strikingly, SCF + G-CSF-repeated treatments robustly and significantly enhanced the corticospinal tract sprouting as compared with the TBI-vehicle controls (Fig. 2b–f, TBI-SCF + G-SCF-repeated vs. TBI-vehicle: C5, C6 and C7, *p* < 0.05; C5-7, *p* < 0.0001). In addition, the number of sprouted corticospinal tract axons at the C6 alone and C5-7 combination in the TBI-SCF + G-CSF-repeated treatment group was also greater than the TBI-SCF + G-CSF-single group (Fig. 2d, C6, *p* < 0.05; Fig. 2f, C5-7, *p* < 0.01). Through correlation analysis, we observed a significantly negative correlation between the number of axons extending to the denervated side (C-5-7) and the latency length of tape removal from the affected forepaw (Fig. 2g, *p* < 0.05, R^2^ = 0.3928), suggesting that the increased axons extending to the denervated side are associated with somatosensory-motor functional improvement.

To validate our findings, we performed sholl analysis to quantify the axon sprouting of the corticospinal tract. The BDA-labeled axons in the left side (i.e. denervated side) of the cervical spinal cord were imaged, and Sholl analysis was performed by plotting the number of intersections against the distance from the central canal of the cervical spinal cord (Fig. 2h). The complexity of the sprouted corticospinal tract in the TBI-vehicle group was significantly increased as compared with the sham group (Fig. 2i–l, *p* < 0.0001). Strikingly, both SCF + G-CSF-single and -repeated treatments robustly enhanced the complexities of sprouted corticospinal tracts as compared with the TBI-vehicle controls (Fig. 2i–l, C5: vehicle vs. SCF + G-CSF-single, *p* < 0.01; vehicle vs. SCF + G-CSF-repeated, *p* < 0.0001. C6, C7 and C5-7: vehicle vs. SCF + G-CSF-single, *p* < 0.0001; vehicle vs. SCF + G-CSF-repeated, *p* < 0.0001). Moreover, the complexity of sprouted corticospinal tract axons in the TBI-SCF + G-CSF-repeated group was also greater than the TBI-SCF + G-CSF-single group at C5 and C6 alone, and C5-7 combination of the cervical spinal cord (Fig. 2i, j, l, *p* < 0.001). Our in vitro study also confirmed that SCF + G-CSF treatment promotes axonal outgrowth (Additional file 3: Fig. S3).

Altogether, our data suggest that SCF + G-CSF treatment in the chronic phase of severe TBI promotes axon regeneration and corticospinal tract sprouting, which is associated with somatosensory-motor  function improvement. SCF + G-CSF-repeated treatments show more robust effects in enhancing corticospinal tract sprouting than the SCF + G-CSF-single treatment.

### The sprouted corticospinal tract axons re-form synaptic structures and are myelinated in the chronic phase of severe TBI

**Fig. 3:**
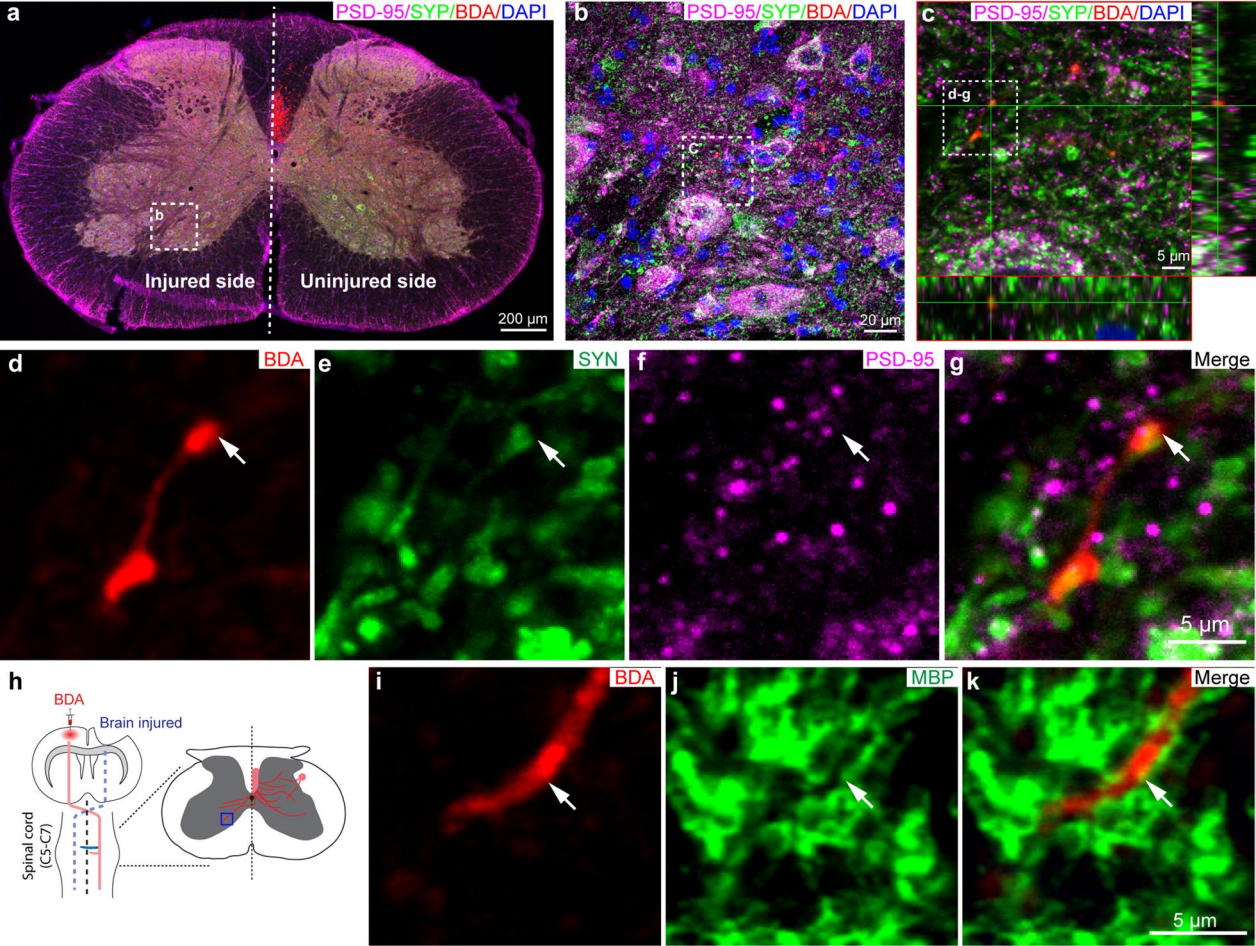
The sprouted intact corticospinal tract axons re-form synaptic structures and are myelinated at the C5-7 segments in the chronic phase of severe TBI. **a**, **b** Representative images show the new synapse formation of the sprouted intact corticospinal tract in the denervated side of cervical spinal cord. The boxed area in the low power image of panel **a** is enlarged in panel **b**. **c** Representative orthogonal view of the newly formed synapses co-expressing BDA, PSD-95 and synaptophysin. Enlarged image of the boxed area shown in panel **b**. The boxed area in panel **c** is enlarged and shown in panels **d**–**g**. **d**–**g** The separated confocal images show a newly formed synapse co-expressing BDA, PSD-95 and synaptophysin (indicated by the arrows). **h** A schematic diagram indicates the imaging area (blue boxed area in the transverse spinal cord) showing the myelin formation in the sprouted corticospinal tract axon projecting from the contralesional cortex to the denervated side of the cervical spinal cord. **i**–**k** The separated confocal images show a sprouted corticospinal tract axon (BDA^+^) that is wrapped by an MBP positive sheath-like structure (indicated by the arrows), indicating that the sprouted corticospinal tract axon is myelinated

We next examined whether the sprouted corticospinal tract axons are able to form synaptic structures and be myelinated. First, we employed two primary antibodies detecting synaptophysin (a pre-synapse marker) and PSD-95 (a post-synapse marker) to co-stain with the BDA-labeled sprouted corticospinal tract axons. As shown in Fig. 3a–g, some of the BDA-labeled axons show bouton-like structures that are  immunopositive to synaptophysin and PSD-95 at the terminal of the BDA-labeled axons, suggesting that the sprouted corticospinal tract axons re-form synapses. We then further examined PSD-95 (excitatory synapse marker) and Gephyrin (inhibitory synapse marker) expression in the denervated side of cervical spinal cord. We observed that Gephyrin expression but not PSD-95 expression was decreased in the TBI-vehicle and TBI-SCF + G-GSF-single treatment groups compared with the sham control group. Importantly, SCF + G-CSF-repeated treatments increased the expression of both PSD-95 and Gephyrin as compared with the TBI-vehicle controls (Additional file 4: Fig. S4). These findings suggest that SCF + G-CSF-repeated treatments enhance synaptic regeneration in the denervated side of cervical spinal cord during the chronic phase of TBI. We further determined the remyelination of the sprouted corticospinal tract axons. We observed that the BDA-labeled sprouted axons were wrapped by myelin basic protein (MBP) (a myelin marker) positive sheath-like structures (Fig. 3h–k), indicating that the sprouted corticospinal tract axons are myelinated.

### SCF + G-CSF treatment in the chronic phase of severe TBI prevents dendritic spine loss in layer 2/3 neurons of the contralateral cortex

**Fig. 4:**
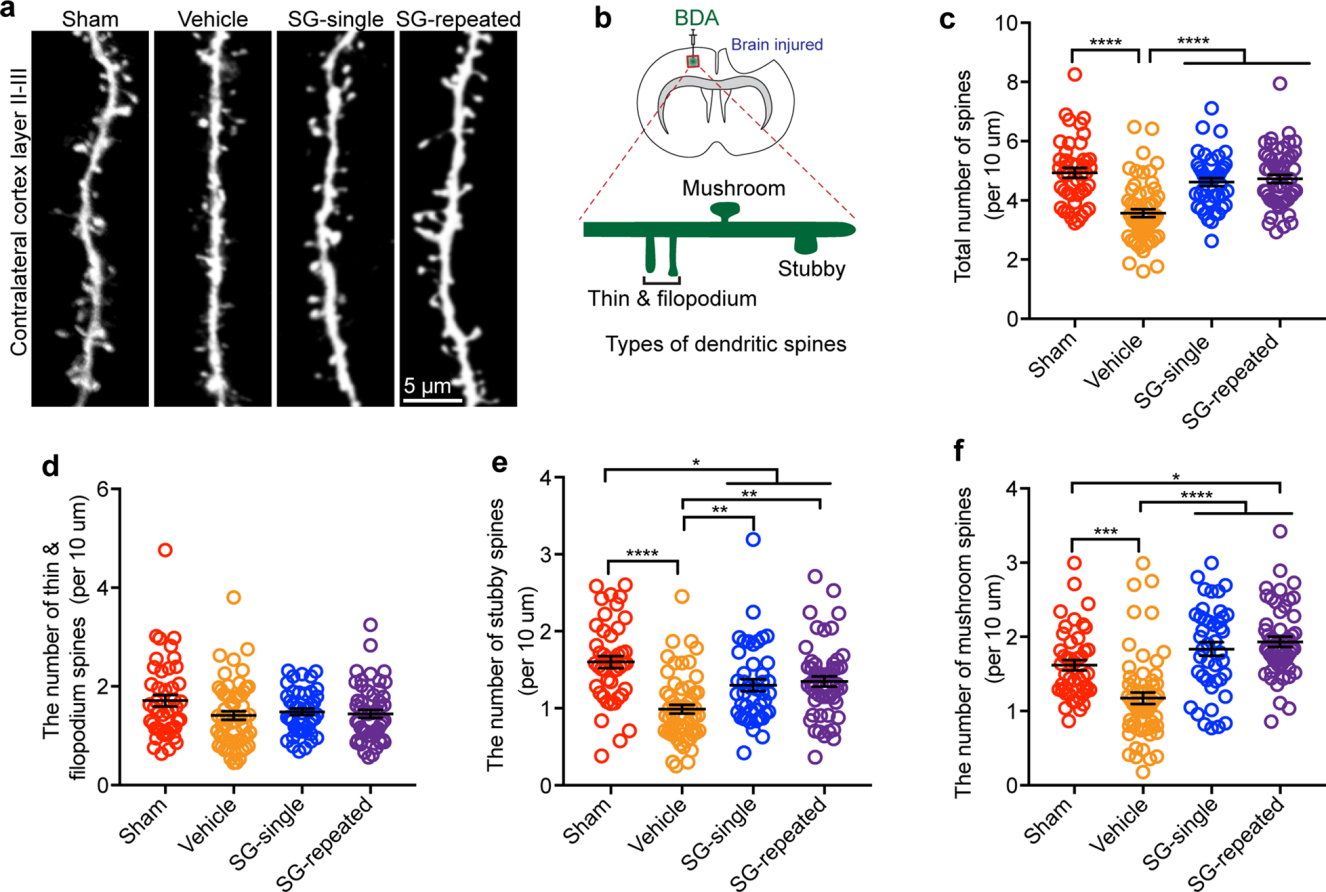
SCF + G-CSF treatment in the chronic phase of severe TBI ameliorates TBI-induced dendritic spine loss in the contralateral cortex layer 2/3 neurons. **a** Representative images show the BDA-labeled dendrites and dendric spines of the contralateral cortex layer 2/3 neurons in different experimental groups. **b** A schematic diagram indicates the location of imaging area and the classification of the dendritic spines. **c-f** The data of quantifying the total number of all-types of spines (**c**), the thin and filopodium spines (**d**), the stubby spines (**e**) and mushroom spines (**f**) on the dendrites. Note that SCF + G-CSF treatment ameliorates TBI-induced spine loss. Each dot in the data graphs represents one dendrite. One-way ANOVA followed by Fisher’s LSD test. Mean ± SEM. *p < 0.05, **p < 0.01, ***p < 0.001, ****p < 0.0001. Sham: n = 45 dendrites (in 4 mice), TBI-vehicle: n = 48 dendrites (in 5 mice), TBI-SCF + G-CSF-single treatment: n = 43 dendrites (in 5 mice), TBI-SCF + G-CSF-repeated treatment: n = 52 dendrites (in 5 mice)

TBI causes dendritic spine loss in both the contralateral and ipsilateral cortex layer 2/3 neurons at 24 h post-injury [79]. However, the long-term effects on dendritic spine changes of the contralateral cortex layer 2/3 neurons in a severe TBI model have not been reported. In the present study, through intracerebral cortical injection of BDA in the contralateral cortex, the dendritic spines of the neurons in the contralateral cortex layer 2/3 were visualized by BDA. As compared with the sham group, the total number of dendritic spines in the TBI-vehicle control group was significantly decreased (Fig. 4a–c, *p* < 0.0001), indicating that severe TBI causes long-term dendritic spine loss in the contralateral cortex in the chronic phase. Both the SCF + G-CSF-single and SCF + G-CSF-repeated treatments prevent the TBI-induced dendritic spine loss (Fig. 4a–c, *p* < 0.0001). No difference was found between the SCF + G-CSF-single and SCF + G-CSF-repeated treatments in preventing dendritic spine loss.

We then classified the spines into three different subtypes: thin and filopodium, stubby and mushroom spines. We observed that the number of thin and filopodium spines had no significant differences among the sham, TBI-vehicle, TBI-SCF + G-CSF-single and TBI-SCF + G-CSF-repeated groups (Fig. 4d). However, the number of stubby spines in the TBI-vehicle group was significantly lower than the sham group (Fig. 4e, *p* < 0.0001). SCF + G-CSF-single and SCF + G-CSF-repeated treatments significantly increased the number of the stubby spines as compared with the TBI-vehicle controls (Fig. 4e, *p* < 0.01), although the number of stubby spines was still less than the sham controls (Fig. 4e, *p* < 0.05). We also determined the change of mushroom type spines. It has been demonstrated that among the types of spines only mushroom spines are stable and functionally active spines [46]. In comparison with the sham group, the number of mushroom spines was significantly decreased in the TBI-vehicle group (Fig. 4f, *p* < 0.001). Both the SCF + G-CSF-single and SCF + G-CSF-repeated treatments significantly increased the number of mushroom spines as compared with the TBI-vehicle controls (Fig. 4f, *p* < 0.0001). Moreover, the number of mushroom spines in the TBI-SCF + G-CSF-repeated treatment group was much greater than in the sham group (Fig. 4f, *p* < 0.05). These findings suggest that severe TBI-induced long-term dendritic loss in layer 2/3 neurons of the contralateral cortex is repaired by SCF + G-CSF treatment in the chronic phase, and that dendritic spine remodeling and mushroom spine formation is also enhanced by the SCF + G-CSF treatment.

### SCF + G-CSF treatment in the chronic phase of severe TBI ameliorates the pathological changes in microglial cells

Microglia play an important role in the pathology and pathological progression of traumatic brain injury. Microglial activation has been reported in the acute and subacute phase of TBI [16, 34, 50]. However, the long-term effects of TBI on microglial changes in the chronic phase still remain unknown.

**Fig. 5:**
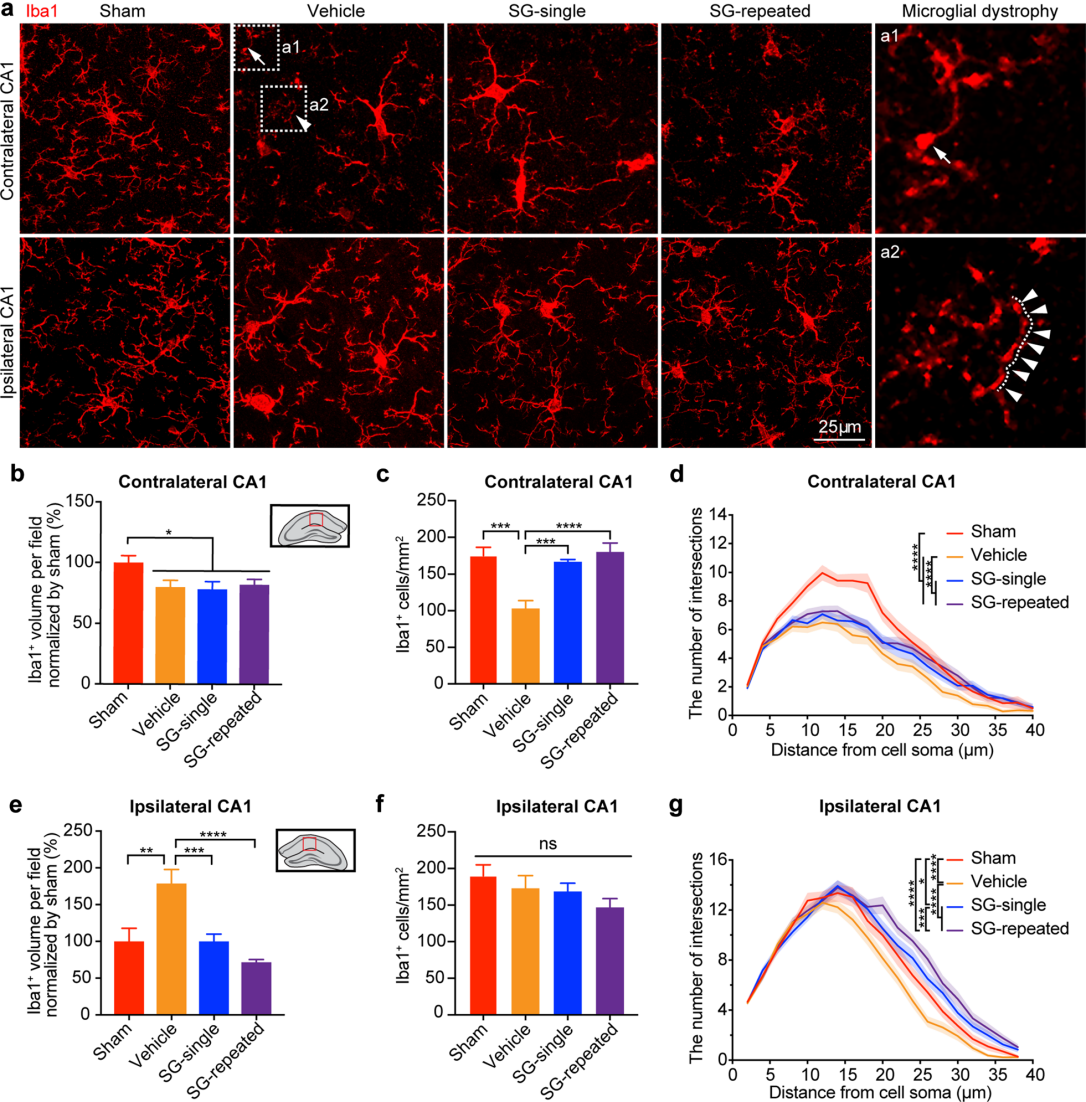
SCF + G-CSF treatment in the chronic phase of severe TBI attenuates TBI-induced microglial pathology in the hippocampus. **a** Representative Z-projection images show Iba1 immunopositive microglia in bilateral hippocampal CA1 in the chronic phase of severe TBI. The boxes of a1 and a2 in TBI-vehicle controls were enlarged to show the detailed morphology of the microglial dystrophy. The white arrow in a1 indicates microglial process beading. The white arrowheads in a2 show microglial process fragmentation. **b** and **e** Quantification data show the alterations of Iba1^+^ microglial volume in the contralateral (**b**) and ipsilateral (**e**) hippocampal CA1. **c** and** f** Quantification data show the number of Iba1^+^ microglia in the contralateral (**c**) and ipsilateral (**f**) hippocampal CA1. Sham: n = 3, TBI-vehicle: n = 4, TBI-SCF + G-CSF-single treatment: n = 5, TBI-SCF + G-CSF-repeated treatment: n = 5. One-way ANOVA followed by Fisher’s LSD test. **d** Sholl analysis data show morphological complexity of microglia in the contralateral hippocampal CA1. **g** Sholl analysis data show morphological complexity of microglia in the ipsilateral hippocampal CA1. In Sholl analysis, Sham: n = 30 microglia (in 3 mice), TBI-vehicle: n = 47 microglia (in 4 mice), TBI-SCF + G-CSF-single treatment: n = 50 microglia (in 5 mice), TBI-SCF + G-CSF-repeated treatment: n = 43 microglia (in 5 mice). Two-way ANOVA followed by Tukey’s post hoc tests. Mean ± SEM. *p < 0.05, **p < 0.01, ***p < 0.001, ****p < 0.0001

In this study, we examined the status of microglia in the chronic phase of TBI. We observed that in the contralateral stratum radiatum of hippocampal CA1, Iba1^+^ cell volume was significantly decreased in all TBI mice treated with or without SCF + G-CSF as compared with the sham mice (Fig. 5a, b, *p* < 0.05). There were no significant differences among the TBI-vehicle, TBI-SCF + G-CSF-single and TBI-SCF + G-CSF-repeated treatment groups (Fig. 5a, b, *p* > 0.05). Moreover, the number of Iba1^+^ microglial cells in the TBI-vehicle group was significantly decreased as compared with sham group (Fig. 5c, *p* < 0.001), and both SCF + G-CSF-single and SCF + G-CSF-repeated treatments rescued the TBI-induced Iba1^+^ microglial loss in the contralateral CA1 (Fig. 5c, *p* < 0.001). Through the Sholl analysis, we observed that the morphological complexity of Iba1^+^ microglial cells in all TBI groups (vehicle, SCF + G-CSF-single and SCF + G-CSF-repeated treatment groups) was significantly reduced when compared with the sham group (Fig. 5d, *p* < 0.0001). SCF + G-CSF treatment increased the morphological complexity of Iba1^+^ microglial cells as compared with the TBI-vehicle controls (Fig. 5d, *p* < 0.0001). In addition, we also observed that some Iba1^+^ microglia with dystrophic morphologies [67, 68] including beaded (Fig. 5a1) and fragmented (Fig. 5a2) microglial processes appeared in the contralateral hippocampus of the TBI-vehicle control mice. These data indicate that microglia in the contralateral hippocampus turn dystrophic in the chronic phase of severe TBI, and that SCF + G-CSF treatment ameliorates the TBI-induced microglial dystrophy.

In the ipsilateral stratum radiatum of hippocampal CA1, the Iba1^+^ cell volume in the TBI-vehicle group was significantly higher than in the groups of sham, TBI-SCF + G-CSF-single and TBI-SCF + G-CSF-repeated treatments (Fig. 5a, e, TBI-vehicle versus sham: *p* < 0.01, TBI-vehicle versus TBI-SCF + G-CSF-single: *p* < 0.001; TBI-vehicle versus TBI-SCF + G-CSF-repeated: *p* < 0.0001). There was no significant difference among sham, TBI-SCF + G-CSF-single and TBI-SCF + G-CSF-repeated treatment groups (Fig. 5a, e, *p* > 0.05). Moreover, no significant differences were observed in the number of Iba1^+^ cells among sham, TBI-vehicle, TBI-SCF + G-CSF-single and TBI-SCF + G-CSF-repeated treatment groups (Fig. 5f, *p* > 0.05). The data of the Sholl analysis showed that the morphological complexity of Iba1^+^ microglial cells in the TBI-vehicle group was significantly reduced as compared with the sham group (Fig. 5g, sham vs. TBI-vehicle: *p* < 0.0001). Similar to the findings from the contralateral hippocampus, SCF + G-CSF treatment significantly increased the morphological complexity of Iba1^+^ microglial cells as compared to the sham and TBI-vehicle controls (Fig. 5g, sham vs. TBI-SCF + G-CSF-single, *p* < 0.05; sham vs. TBI-SCF + G-CSF-repeated, *p* < 0.0001; TBI-vehicle vs. TBI-SCF + G-CSF-single, *p* < 0.0001; TBI-vehicle vs. TBI-SCF + G-CSF-repeated, *p* < 0.0001). In addition, SCF + G-CSF-repeated treatments significantly enhanced the morphological complexity of Iba1^+^ microglial cells when compared to the SCF + G-CSF-single treatment (Fig. 5g, *p* < 0.001). These data suggest that the microglia in the ipsilateral hippocampus display an activated phenotype with increased cell volume and reduced processes. SCF + G-CSF treatment enhances the transformation of Iba1^+^ microglial cells from the activated phenotype into the homeostatic phenotype demonstrated by reduced cell volume and increased processes in the chronic phase of severe TBI.

**Fig. 6:**
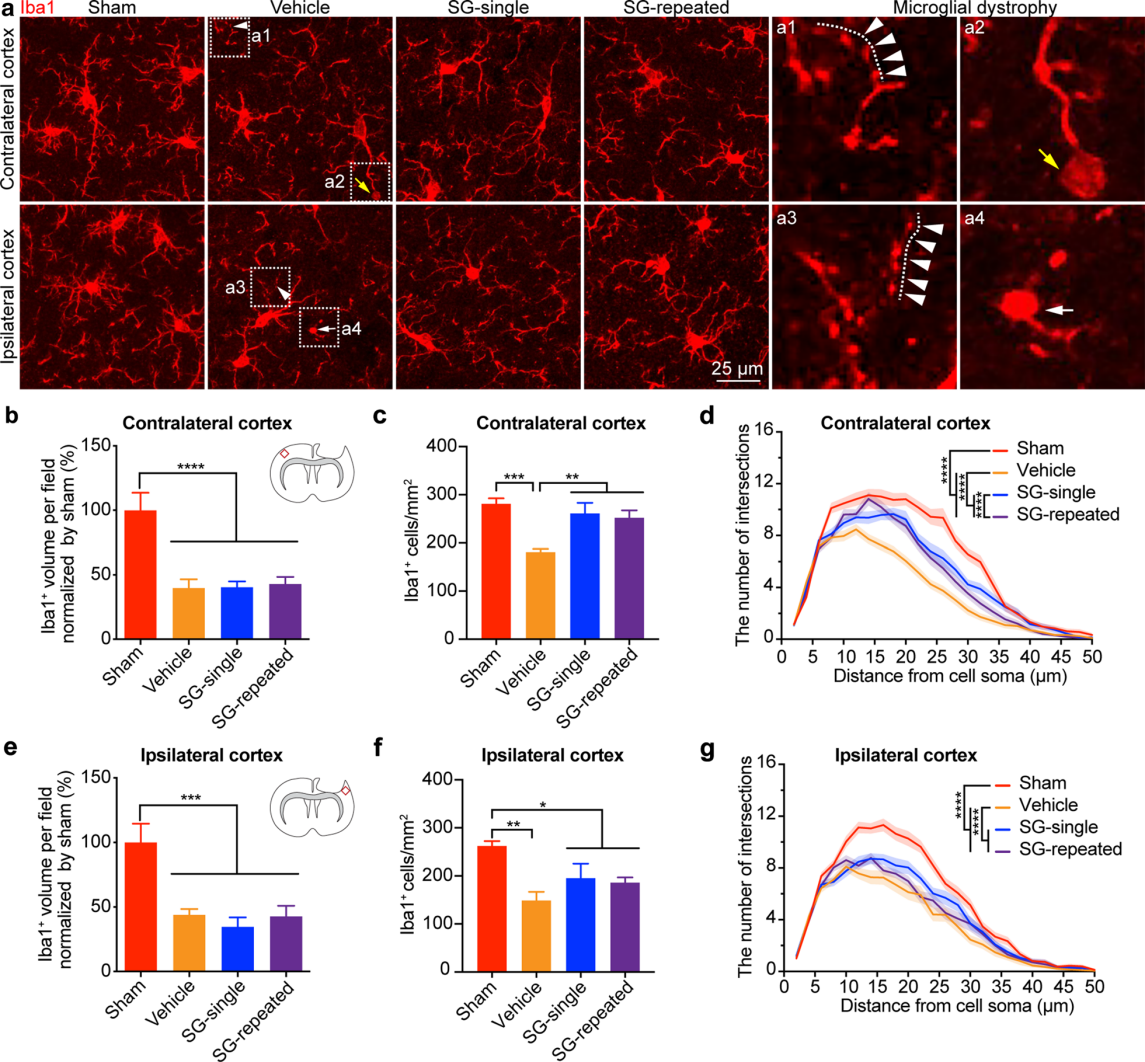
SCF + G-CSF treatment in the chronic phase of severe TBI attenuates TBI-induced microglial pathology in the cortex. **a** Representative z-projection images show Iba1 immunopositive microglia in bilateral cortex in the chronic phase of TBI. The boxes of a1–a4 in TBI-vehicle controls were enlarged to show the detailed morphology of the microglial dystrophy. The white arrowheads in a1 and a3 show microglial process fragmentation. The yellow arrow in a2 indicates a large swelling (spheroid) at the end of microglial process. The white arrow in a4 shows microglial process beading. **b** and **e** Quantification data show that the volume of Iba1^+^ microglia is reduced in all TBI groups (TBI-vehicle, TBI-SCF + G-CSF-single treatment and TBI-SCF + G-CSF-repeated treatment groups) in both the contralateral (**b**) and ipsilateral (**e**) cortex. **c** and **f** Quantification data show the number of Iba1^+^ microglia in the contralateral (**c**) and ipsilateral (**f**) cortex. Note that the TBI-reduced Iba1^+^ microglia are restored by SCF + G-CSF treatment in the contralateral but not in the ipsilateral cortex. Sham: n = 3, TBI-vehicle: n = 4, TBI-SCF + G-CSF-single treatment: n = 5, TBI-SCF + G-CSF-repeated treatment: n = 5. One-way ANOVA followed by Fisher’s LSD test. **d** Sholl analysis data show morphological complexity of microglia in the contralateral cortex. Sham: n = 36 microglia (in 3 mice), TBI-vehicle: n = 43 microglia (in 4 mice), TBI-SCF + G-CSF-single treatment: n = 43 microglia (in 5 mice), TBI-SCF + G-CSF-repeated treatment: n = 39 microglia (in 5 mice). **g** Sholl analysis data show morphological complexity of microglia in the ipsilateral cortex. Note that TBI-reduced morphological complexity of microglia in both the contralateral and ipsilateral cortex is ameliorated by SCF + G-CSF treatment. Sham: n = 41 microglia (in 3 mice), TBI-vehicle: n = 40 microglia (in 4 mice), TBI-SCF + G-CSF-single treatment: n = 38 microglia (in 5 mice), TBI-SCF + G-CSF-repeated treatment: n = 40 microglia (in 5 mice). Two-way ANOVA followed by Tukey’s post hoc test for Sholl analysis. Mean ± SEM. *p < 0.05, **p < 0.01, ***p < 0.001, ****p < 0.0001

We also examined the microglial morphology in both the contralateral and ipsilateral cortex. The Iba1^+^ cell volume in the bilateral cortex was significantly decreased in all TBI groups (i.e.TBI-vehicle, TBI-SCF + G-CSF-single and TBI-SCF + G-CSF-repeated treatment groups) when compared with the sham group (Fig. 6a, b, e, contralateral, *p* < 0.0001; ipsilateral, *p* < 0.001). In both the contralateral and ipsilateral cortex, the number of Iba1^+^ microglial cells was significantly decreased in the TBI-vehicle group as compared with the sham group (Fig. 6c, contralateral, *p* < 0.001; ipsilateral, *p* < 0.01). In the contralateral cortex, both the SCF + G-CSF-single and SCF + G-CSF-repeated treatments rescued the TBI-induced microglial loss (Fig. 6c, *p* < 0.01), while this beneficial effect was not seen in the ipsilateral cortex (Fig. 6f). The findings of the Sholl analysis showed that the morphological complexity of the Iba1^+^ microglia in the bilateral cortex was reduced in the TBI-vehicle group as compared with the sham group (Fig. 6d, g, *p* < 0.0001). SCF + G-CSF treatment significantly increased the morphological complexity of the Iba1^+^ microglial cells in the bilateral cortex when compared with the TBI-vehicle controls (Fig. 6d, g, *p* < 0.0001), while the levels of the SCF + G-CSF-enhanced morphological complexity were still lower than the sham controls (Fig. 6d, g, *p* < 0.0001). In the contralateral cortex, SCF + G-CSF-repeated treatments led to significant increases in the morphological complexity of Iba1^+^ microglial cells as compared to the SCF + G-CSF-single treatment (Fig. 6d, *p* < 0.0001). Furthermore, we also observed that some microglia in the bilateral cortex of the TBI-vehicle control mice had dystrophic characteristics including fragmentation of cytoplasmic processes (Fig. 6a1, a3) as well as large spheroidal swelling (Fig. 6a2) and beading (Fig. 6a4) at the end of the processes. Together, our findings suggest that widespread microglial phathology with reduced volume, number, and processes as well as showing dystrophic features occurs in the bilateral cortex during the chronic phase of severe TBI, and that SCF + G-CSF treatment in the chronic phase of severe TBI ameliorates the TBI-induced microglial phathology and increases microglial processes in both the contralateral and ipsilateral cortex.

### SCF + G-CSF treatment in the chronic phase of severe TBI promotes synapse re-balance in the hippocampus

**Fig. 7:**
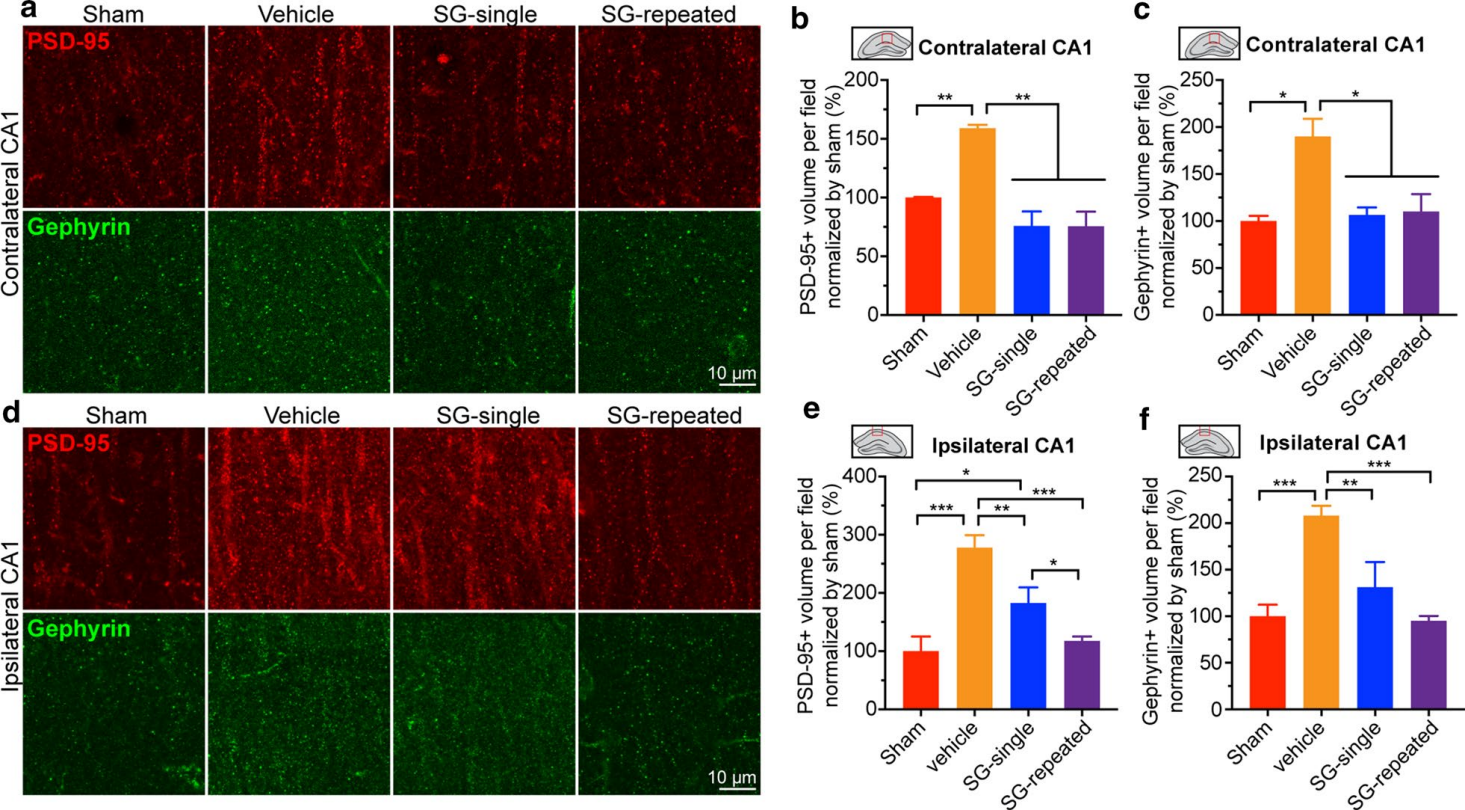
TBI-induced synaptic overgrowth in the bilateral hippocampal CA1 is prevented by SCF + G-CSF treatment in the chronic phase of severe TBI. **a** Representative single layer of z-stack images shows PSD-95 and Gephyrin immunopositive synapses in the contralateral hippocampal CA1. **b**, **c** Quantification data show the volumes of PSD-95 (**b**) and Gephyrin (**c**) immunopositive synapses in the contralateral hippocampal CA1. **d** Representative single layer of z-stack images shows PSD-95 and Gephyrin immunopositive synapses in the ipsilateral hippocampal CA1. **e**, **f** Quantification data show the volumes of PSD-95 (**e**) and Gephyrin (**f**) immunopositive synapses in the ipsilateral hippocampal CA1. The red boxes in panels **b**, **c**, **e** and **f** indicate the imaging areas in the bilateral hippocampal CA1 regions for data analysis. One-way ANOVA followed by Fisher’s LSD test. Mean ± SEM. *p < 0.05, **p < 0.01, ***p < 0.001. Sham: n = 3, TBI-vehicle: n = 4, TBI-SCF + G-CSF-single treatment: n = 5, TBI-SCF + G-CSF-repeated treatment: n = 5

TBI causes hippocampal synapse loss in the acute phase, which starts to recover in the subacute phase, but the recovery is incomplete as the number of hippocampal synapses is still lower than in naive mice [23, 41, 63]. However, the long-term effects of TBI on synaptic changes in the brain remain unexplored. In the present study, we determined the severe TBI-induced synaptic changes in both the cortex and hippocampus by immunostaining with PSD-95 and Gephyrin antibodies in the chronic phase. In both the contralateral and ipsilateral cortex, the volumes of PSD-95^+^ and Gephyrin^+^ synapses did not show significant differences among sham, TBI-vehicle, TBI-SCF + G-CSF-single and TBI-SCF + G-CSF-repeated treatment groups (Additional file 5: Fig. S5, a–f, *p *> 0.05). However, in both the contralateral and ipsilateral stratum radiatum of hippocampal CA1, the volumes of both the PSD-95^+^ and Gephyrin^+^ synapses in the TBI-vehicle group were significantly increased as compared with the sham group (Fig. 7a–c, contralateral: PSD-95, *p* < 0.01, Gephyrin, *p* < 0.05; Fig. 7d–f, ipsilateral: PSD-95, *p* < 0.001, Gephyrin, *p* < 0.001), suggesting that the severe TBI-induced overgrowth of synapses occurs in the hippocampus in the chronic phase. Strikingly, in the contralateral stratum radiatum of hippocampal CA1, both the SCF + G-CSF-single and SCF + G-CSF-repeated treatments significantly reduced PSD-95^+^ and Gephyrin^+^ synapses when compared with the TBI-vehicle controls (Fig. 7a–c, PSD-95: *p* < 0.01; Gephyrin: *p* < 0.05). There was no significant difference between SCF + G-CSF-treated groups and sham group (Fig. 7a–c, sham vs. TBI-SCF + G-CSF-single and TBI-SCF + G-CSF-repeated treatments, *p* > 0.05). These data indicate that severe TBI-induced overgrowth of synapses in the contralateral hippocampal CA1 is completely prevented by SCF + G-CSF treatment in the chronic phase. Similar data were observed in the ipsilateral hippocampal CA1 region (Fig. 7d–f), except the PSD-95 expression in the TBI-SCF + G-CSF-single treatment group showed significantly higher levels than the sham and TBI-SCF + G-CSF-repeated treatment group (Fig. 7d, e, *p* < 0.05).

We also analyzed the number of neurons in both the contralateral and ipsilateral hippocampal CA1. We observed that there were  no significant differences among sham, TBI-vehicle, TBI-SCF + G-CSF single treatment and TBI-SCF + G-CSF repeated treatment groups (Additional file 6: Fig. S6), suggesting that severe TBI does not lead to neuron loss in the bilateral hippocampal CA1 in the chronic phase. SCF + G-CSF treatment in the chronic phase of TBI does not change neuron numbers in the bilateral hippocampal CA1. These findings demonstrate that severe TBI-caused overgrowth of synapses and SCF + G-CSF treatment-rebalanced synapses in the bilateral hippocampal CA1 in the chronic phase are associated with the alterations of synaptic formation but not linked to neuronal number change.

### SCF + G-CSF treatment enhances pruning of synapses by microglia in both in vivo and in vitro

Microglial cells express both SCF receptor and G-CSF receptor [2, 82, 84], supporting the possibility of SCF and G-CSF in regulating the function of microglia [2, 72]. It has been shown that microglial cells play an important role in pruning synapses in both neuronal development and diseases [32, 33, 43, 78]. Therefore, we hypothesized that SCF + G-CSF may enhance the ability of microglial cells to prune the TBI-induced overgrown synapses in the hippocampus.

**Fig. 8:**
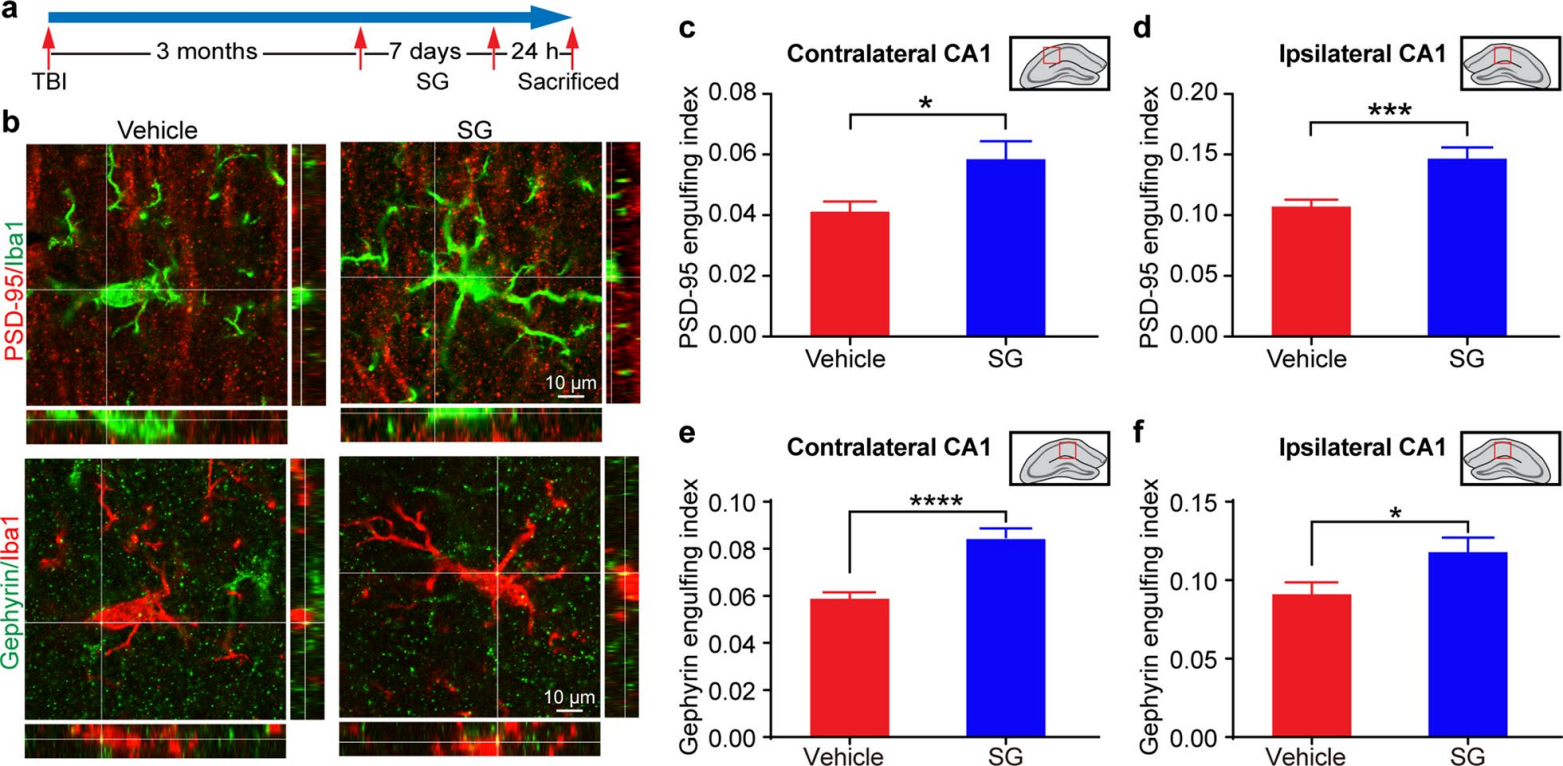
SCF + G-CSF treatment in the chronic phase of severe TBI enhances pruning of synapses by microglia in the hippocampal CA1. **a** Experimental flowchart. **b** Representative orthogonal views of confocal images show that Iba1 positive microglia engulf PSD-95 and Gephyrin positive synapses. **c**–**f** Quantification data show that SCF + G-CSF treatment enhances microglia in engulfing PSD-95 (**c**, **d**) and Gephyrin (**e**, **f**) positive synapses in both the contralateral (**c**, **e**) and ipsilateral (**d**, **f**) hippocampal CA1. The red boxes in panels **c**–**f** indicate the imaging areas in the bilateral hippocampal CA1 regions for data analysis. Student’s t test. Mean ± SEM. *p < 0.05, ***p < 0.001, ****p < 0.0001. TBI-vehicle: n = 24 microglia (in 4 mice), TBI-SCF + G-CSF: n = 30 microglia (in 5 mice)

To test this hypothesis, we carried out a new experiment to examine the efficacy of SCF + G-CSF treatment in the chronic phase of severe TBI in engulfing synapses in the hippocampus. In this experiment, mice were sacrificed 24 h after a 7-day treatment of SCF + G-CSF initiated 3 months after severe TBI (Fig. 8a). We then analyzed the microglial cell-engulfed synapses in the hippocampus. In both the contralateral and ipsilateral hippocampal CA1 regions, the capacity of Iba1^+^ microglial cell-engulfed PSD-95 and Gephyrin positive synapses was significantly increased by SCF + G-CSF treatment (Fig. 8b–f, Contralateral: engulfed PSD-95, *p* < 0.05; engulfed Gephyrin, *p* < 0.0001. Ipsilateral: engulfed PSD-95, *p* < 0.001; engulfed Gephyrin, *p* < 0.05). Since both resident microglia and monocyte-derived macrophages express Iba1, in order to validate the findings observed from Iba1 positive cells, we also used a specific marker for resident microglia, P2RY12, to specifically label the resident microglial cells. We observed similar results as shown in Iba1 positive cells (Additional file 7: Fig. S7). These data suggest that SCF + G-CSF treatment in the chronic phase of severe TBI enhances the function of microglia in pruning synapses in the hippocampus.

**Fig. 9:**
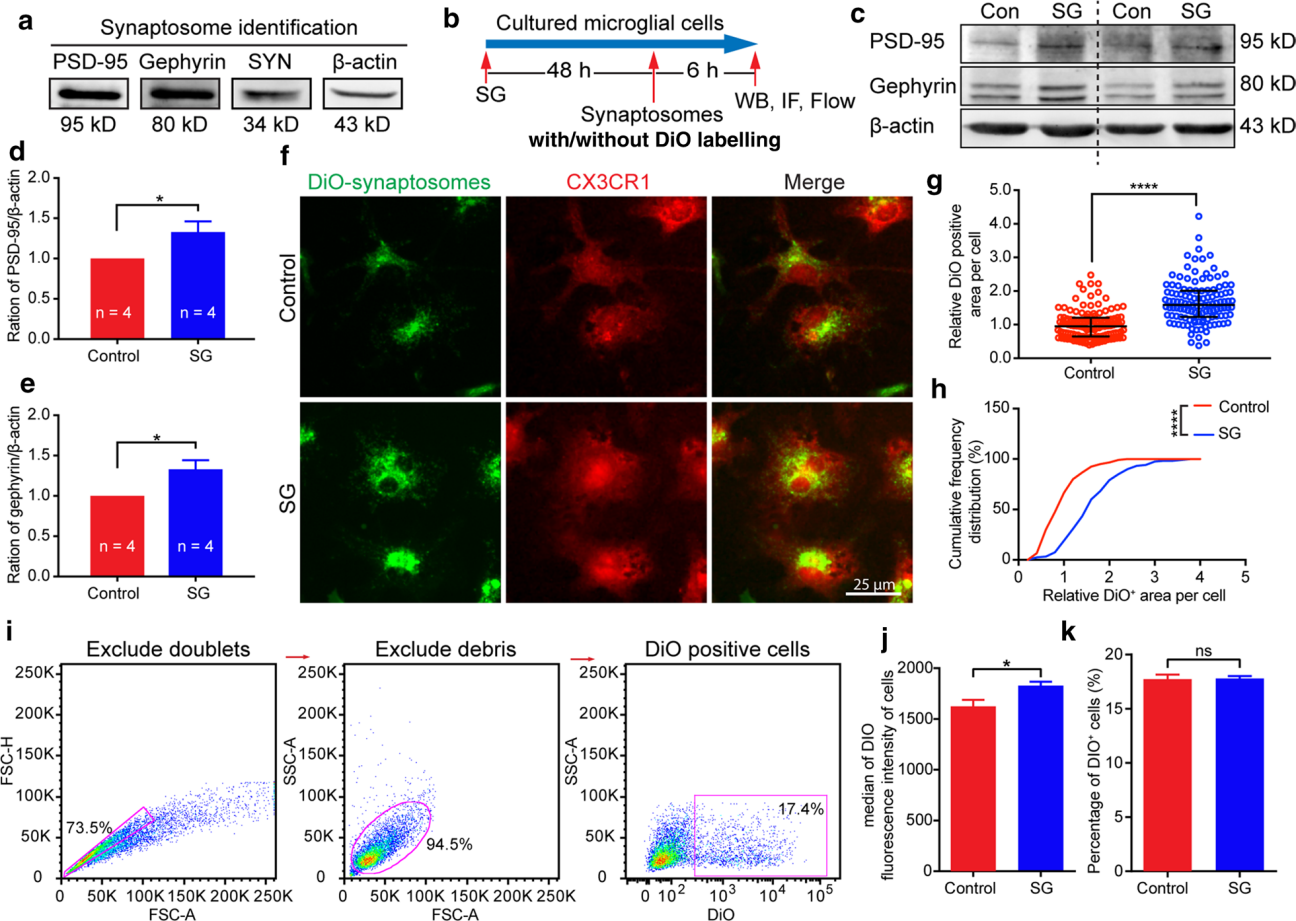
SCF + G-CSF treatment enhances microglia in engulfing synaptosomes in vitro. **a** Western blotting shows that isolated synaptosomes express synaptic proteins. **b** A schematic flowchart of the in vitro study. **c** Representative western blot images show that PSD-95 and Gephyrin are expressed in the cultured microglia after adding synaptosomes. **d** and **e** Quantification data of western blots show that the levels of PSD-95 (**d**) and Gephyrin (**e**) in the cultured microglia are increased by SCF + G-CSF treatment. Control: n = 4, SCF + G-CSF treatment: n = 4. **f** Representative immunocytochemistry images show the engulfed DiO-labeled synaptosomes (green) in cultured microglia (red, CX3CR1 positive). **g** The scatterplot bar graph shows that SCF + G-CSF treatment increases uptake of synaptosomes (DiO positive area) by the cultured microglia. Each dot represents a single microglial cell. Control: n = 60 microglia, SCF + G-CSF treatment: n = 60 microglia. Data were collected from four independent experiments. **h** The cumulative frequency distribution of DiO positive area in microglial cells. Note that the engulfed synaptosomes (DiO positive area) by the cultured microglia are increased by SCF + G-CSF treatment. Kolmogorov–Smirnov test. **i** Representative scatterplot graphs of flow cytometry show the strategy of data analysis. The doublets and debris are excluded, and only the DiO positive cells are analyzed. **j** The bar graph shows the DiO fluorescence density in microglial cells. **k** The bar graph shows the percentage of DiO positive microglial cells. Control: n = 3, SCF + G-CSF treatment: n = 3. Student’s t test. Mean ± SEM. *p < 0.05, ****p < 0.0001

We also used an in vitro approach to further validate the in vivo data. Before performing the experiment, we confirmed that the purity of cultured primary microglia was 97.8% (Additional file 8: Fig. S8). Synaptosomes were isolated from adult mouse cortex and confirmed using specific synaptic markers. The western blot data showed that the isolated synaptosomes expressed specific synaptic markers: PSD-95, Gephyrin and synaptophysin (Fig. 9a). We then added the synaptosomes into microglia with/without SCF + G-CSF treatment for 48 h. The western blot data revealed that both engulfed PSD-95 and Gephyrin in the microglia were significantly increased after SCF + G-CSF treatment (Fig. 9b–e and Additional file 9: Fig. S9, *p* < 0.05), supporting that SCF + G-CSF enhances the ability of microglia in engulfing synaptosomes in vitro.

The immunocytochemistry data further confirmed the findings of western blot. Synaptosomes were labeled with DiO to track their uptake by microglial cells. We found that the DiO positive area in microglial cells was significantly increased by SCF + G-CSF treatment (Fig. 9f–h, *p* < 0.0001), indicating that SCF + G-CSF treatment reinforces the function of microglia in engulfing synaptosomes. Moreover, we also used a flow cytometry approach to further validate our findings. We observed that the median fluorescence intensity of DiO-labeled synaptosomes per microglial cell was significantly increased by SCF + G-CSF treatment (Fig. 9i, j, *p* < 0.05). However, the percentage of DiO positive cells did not show significant difference between SCF + G-CSF treatment and control group (Fig. 9i, k, *p* > 0.05), demonstrating that SCF + G-CSF enhances the ability of microglia to engulf the synaptosomes.

In total, both in vivo and in vitro studies confirmed that SCF + G-CSF treatment reinforces the function of microglia in engulfing synapses.

## Discussion

**Fig. 10:**
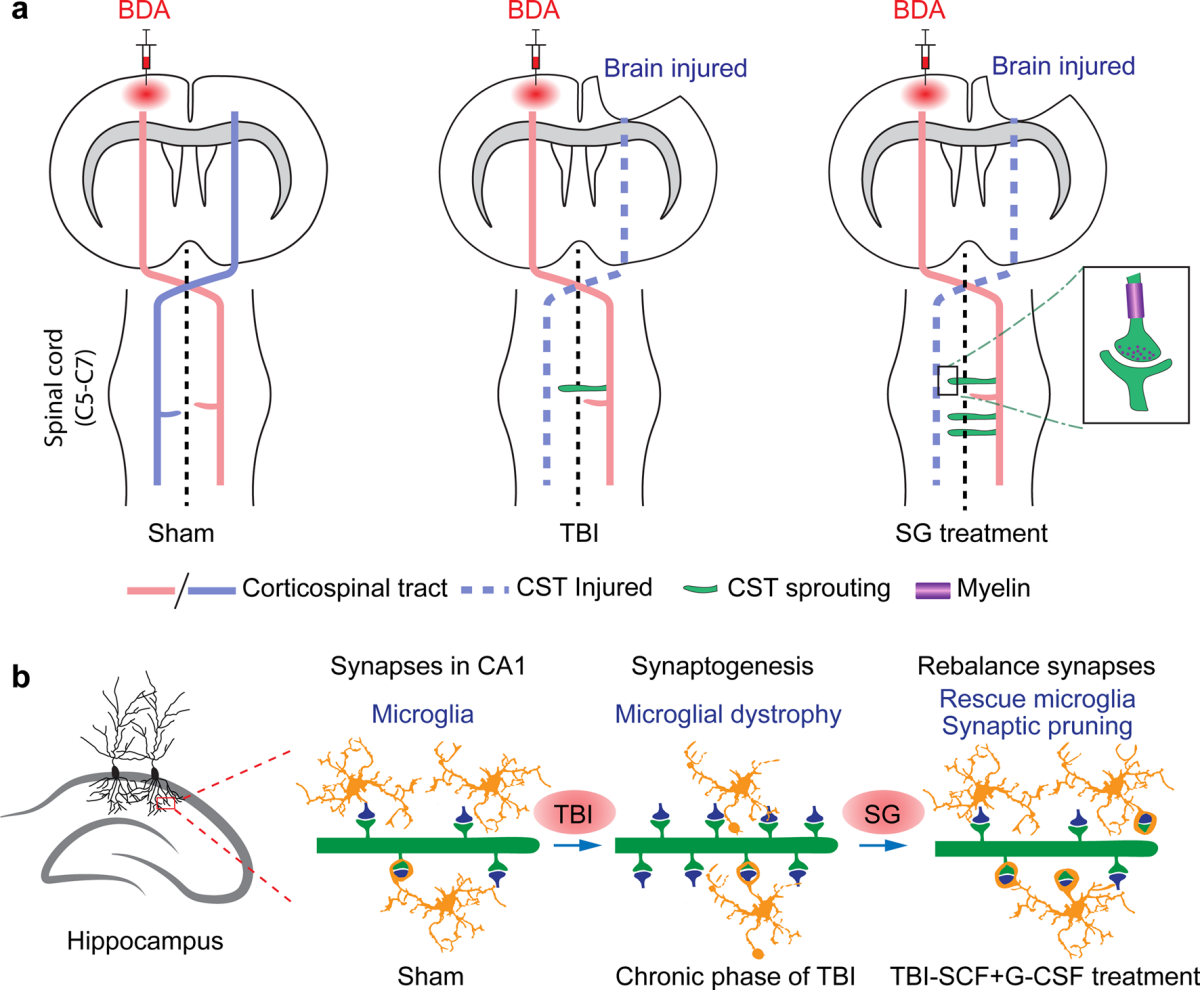
Schematic diagrams describe the potential mechanisms of the SCF + G-CSF-enhanced neurorestoration in the chronic phase of severe TBI. **a** SCF + G-CSF treatment in the chronic phase of severe TBI promotes intact corticospinal tract sprouting into the denervated side of the cervical spinal cord (C5-7). The sprouted corticospinal tract axons are myelinated and re-form synaptic connections. **b** SCF + G-CSF treatment in the chronic phase of severe TBI ameliorates the TBI-induced microglial pathology and rebalances the TBI-induced synaptic overgrowth in the hippocampus by enhancing microglial function in pruning the over-generated synapses

The main findings of this study are summarized in Fig. 10. The data of the present study reveal that systemic administration of SCF + G-CSF in the chronic phase of severe TBI improves somatosensory-motor function and spatial learning and memory. Our findings also show that the potential mechanism underlying the SCF + G-CSF-improved neurological outcome is associated with enhancing contralateral corticospinal tract (CST) sprouting into the denervated side of the cervical spinal cord forming synapses for reinnervation and reinforcing removal of the overgrown synapses in the hippocampus by microglia. To the best of our knowledge, the current study provides the first evidence supporting that SCF + G-CSF treatment in the chronic phase of severe TBI enhances CST sprouting. Moreover, in this study we have identified a unique pathology of microglia in morphological changes (i.e. microglial dystrophy) that occurs in the chronic phase of severe TBI and demonstrated therapeutic efficacy of SCF + G-CSF treatment in the chronic phase in ameliorating the severe TBI-caused persistent microglial pathology  in both the cortex and hippocampus. As compared with single time treatment, SCF + G-CSF-repeated treatments show better restorative efficacy in the chronic phase of severe TBI.

The therapeutic time window is a crucial element for drugs to treat TBI [49]. Most preclinical and clinical studies focus on treatment in the acute phase of TBI (within 24 h), and many drugs lose efficacy after delayed treatment [49]. Increasing evidence has shown that TBI is a long-term progressive disease [3, 10, 35]; however, no effective treatment is available for the chronic phase of TBI. It has been demonstrated that both SCF and G-CSF can pass through the blood–brain barrier under normal physiological conditions [65, 86]. Our previous study has shown that SCF + G-CSF combination treatment in the chronic phase of TBI (3 months post injury) improves severe TBI-impaired spatial learning/memory and somatosensory motor function as compared with SCF and G-CSF alone treatment [57]. In the present study, similar results have been found in the SCF + G-CSF-repeated treatments. We have noticed slight improvement of spatial learning and memory in the water maze test after SCF + G-CSF-repeated treatments (i.e. significant change is seen at day 4 of the test). This finding appears not to be in line with the robust reparative effects of SCF + G-CSF-repeated treatments in restoration of synapse balance in the hippocampus where severe TBI causes overgrown synapses in the chronic phase. There are several reasons that may negatively affect the findings of water maze test in this study. It is worth noting that in this study we reversed the light/dark cycle in the animal facility and performed the behavioral tests during the active phase of the mice under red light (i.e. daytime for human). This artificial light/dark cycle switch may affect the sensitivity of water maze testing because the red light decreases visual sensitivity in mice [56]. Moreover, our data show persistent impairments of motor function in the chronic phase of severe TBI. We performed the water maze test in TBI mice (C57BL mice) at the age of ~ 11 months. Both motor function impairment and testing of water maze in middle aged C57BL mice (11–12 months) have been shown to negatively affect the results of water maze test [5, 15, 39]. Timing for taking water maze test after treatment may affect the findings. The findings of the current study reveal that the SCF + G-CSF-single treatment does not show significant improvement in the water maze test as compared with the TBI-vehicle controls, which is not in line with our previous study [57]. It is worth mentioning that the water maze test performed in the present study is 10 weeks delayed as compared with our previous study, suggesting that the progressive impairments of spatial learning and memory in the chronic phase of severe TBI may cover the single SCF + G-CSF treatment-induced improvement. The SCF + G-CSF-repeated treatments may be necessary for long-term functional recovery in the chronic phase of severe TBI.

Substantial evidence has shown that neuronal network reorganization in the peri-injury cortex and the intact cortex remote to the lesion area plays an important role in functional improvement after brain injury [1, 14, 28, 53]. The TBI model used in the current study causes severe damage in the somatosensory motor cortex of the right hemisphere, which may lead to a limitation of the compensatory recovery from the peri-injury region. Therefore, we seek to examine neurostructural changes in the contralateral cortex. Strikingly, even when SCF + G-CSF treatment is given at 3 months post-TBI, robust regenerative growth of the intact CST axons projecting from the contralesional cortex is detected in the denervated side of the cervical spinal cord at the C5-7 spinal segments which contain neurons/nerve fibers controlling somatosensory-motor function in the shoulder, forearm and hand/paw. The sprouted CST axons are myelinated and form synaptic connections with spinal cord neurons. SCF + G-CSF-repeated treatments enhance synaptic regeneration in the denervated side of the cervical spinal cord. Importantly, our findings also reveal that the numbers of sprouted CST axons at C5-7 spinal segments are positively correlated with somatosensory-motor function recovery. These findings suggest that SCF + G-CSF-enhanced compensatory neurostructural regeneration in the contralesional cortex-derived CST contributes to somatosensory-motor function improvement  in the chronic phase of severe TBI. The mechanism underlying the SCF + G-CSF-enhanced CST sprouting remains unclear. Our previous studies have demonstrated that SCF + G-CSF treatment has a direct effect in promoting neurite outgrowth in cultured cortical neurons through MEK/ERK/p53 and PI3K/AKT/NF-kB/brain-derived neurotrophic factor (BDNF) signal pathways [22, 69]. The NF-kB/BDNF signaling pathway has been shown to be crucial for intact CST sprouting following brain injury [75].

Dendritic degeneration has been proposed to be associated with functional impairments following TBI [81]. The neurons in cortical layer 2–3 are a direct source of excitatory input to cortical layer 5 pyramidal neurons and control the gain of layer 5 neurons [42, 59] that directly connect motor subcortical structures to drive movement [17, 55]. A previous study has shown that TBI causes dendritic spine loss in both the contralateral and ipsilateral cortex (layer 2–3) and hippocampus (dentate gyrus) in the acute phase of TBI (at 24 h post-injury) [79]. The findings of the present study provide new evidence showing that severe TBI leads to long-term dendritic spine loss in the contralateral cortex (layer 2–3) in the chronic phase. Our data have also demonstrated that SCF + G-CSF treatment in the chronic phase of severe TBI prevents the TBI-induced dendritic spine loss. How SCF + G-CSF treatment prevents the dendritic spine loss in the chronic phase of severe TBI remains an open question. It could be possible that the signal pathways that are required for SCF + G-CSF-enhanced neurite extension of cortical neurons may also play a role in SCF + G-CSF-attenuated dendritic spine loss. Although the dendritic spine loss is observed on the individual dendrites of neurons in the contralateral cortex layer 2/3 in the chronic phase of severe TBI, the expression of synaptic markers (PSD-95 and Gephyrin) in the contralateral cortex is unchanged. It has been proposed that the spine loss of the individual dendrites may be accompanied by compensatory dendritic increase in the contralateral hemisphere after brain injury [37]. Being in line with this postulation, our previous findings have shown that the MAP2 positive dendrites in the contralateral cortex are not changed in the chronic phase of severe TBI [57].

Our data show that in the chronic phase of severe TBI, the increased expression of synaptic markers (Gephyrin and PSD-95) is seen only in the bilateral hippocampal CA1, but not in the cortex and CST-denervated side of the spinal cord. The discrepancy of these findings may be attributed to different pathological progressions in different regions in the chronic phase of severe TBI. The neurons in layer 2/3 of the contralateral cortex directly connect with the neurons in the ipsilateral hemisphere [38], and Wallerian degeneration occurs in the damaged axons [30]. The spinal cord neurons encounter anterograde transneuronal degeneration after TBI, which could induce synaptic loss in the spinal cord. The hippocampal neurons directly connect with the entorhinal cortex [6] which is not directly injured in the TBI model used in the present study. These different connecting networks within ipsilesional cortex may cause the different pathological progression in the chronic phase of severe TBI.

Reactive synaptogenesis has been demonstrated after TBI [36, 63]. A time-course study has revealed that about 60% of synapses are lost in hippocampal CA1 at 2 days post-injury; the synapses are increased to ~ 75% of pre-injury levels 60 days after TBI [63]. It has been shown that the synaptophysin expressing synapses in the hippocampus are significantly increased 24 weeks, but not 12 weeks, after TBI in a mouse model of Alzheimer’s disease [87], suggesting a delayed reaction of synaptogenesis after TBI. Our data demonstrate that both the excitatory (PSD-95 positive) and inhibitory (Gephyrin positive) synapses in both the contralateral and ipsilateral hippocampal CA1 are significantly increased at 12 months post-severe TBI as compared with the sham controls, indicating massive synaptogenesis occurring in the hippocampus during the chronic phase of severe TBI. This observation is consistent with our earlier study [57].

Severe TBI leads to a complex synaptic pathology in the chronic phase. The reparative effects of SCF + G-CSF treatment in the chronic phase of TBI are dependent on different synaptic pathologies in the cortex, hippocampus and spinal cord. In the contralateral cortex and the denervated cervical spinal cord, decreased dendritic spines in the contralesional cortex and decreased synapses in the denervated cervical spinal cord are restored by SCF + G-CSF treatment (especially the repeated treatments). However, in the bilateral hippocampal CA1 regions, TBI-caused overgrowth of synapses is prevented by SCF + G-CSF treatment (especially the repeated treatments). The overgrowth of synapses in the hippocampal CA1 may activate the microglial cell-regulated synaptic pruning to maintain the synaptic functional balance [48]. Impaired microglial function in synaptic pruning may contribute to TBI-caused overgrown synapses in the hippocampal CA1. SCF + G-CSF treatment may ameliorate the pathological status of microglia and enhance synaptic pruning to keep synaptic balance in the hippocampal CA1.

The findings of the present study reveal that microglia appear to have different pathological reactions in the cortex and hippocampus of the contralateral and ipsilateral hemispheres in the chronic phase of severe TBI. Microglia have an activated phenotype in the ipsilateral hippocampus, while in the contralateral hippocampus and bilateral cortex microglia show dystrophic degeneration in the TBI vehicle control mice. The different pathological reactions of microglia in the bilateral cortex and hippocampus reflect that the pathological microenvironment of these brain regions is different due to an unevenly progressed pathological condition in the brain regions during the chronic phase of severe TBI. In addition to showing microglial dystrophy, we have also observed that the number of microglia is decreased in the contralateral hippocampus and bilateral cortex in the TBI vehicle control mice, suggesting that the decreased microglia could be the result of dystrophic degeneration-induced microglial loss that occurs in the chronic phase of severe TBI. It is worth noting that the dystrophic Iba1 positive microglia showing loss of branches and appearance of beading, fragmentation and large swelling (spheroid) at the end of the process which we observed in the brains of severe TBI mice in the chronic phase, have previously been identified in the brains of neurodegenerative diseases including Alzheimer’s disease [68]. These dystrophic (senescent) microglia with impaired microglial function and responses have been suggested to insufficiently support neuronal function and play essential roles in the development and progression of neurodegenerative diseases. Recent clinical studies have demonstrated that severe TBI increases the risk of Alzheimer’s disease-like dementia which remains more than 30 years after TBI [31, 52]. In the present study, we have discovered the existence of dystrophic (senescent) microglia in the contralateral hippocampus and bilateral cortex in the chronic phase of severe TBI. The widespread dystrophic (senescent) microglia in the brain during chronic phase of severe TBI could impair brain function due to microglial dystrophy-induced dysfunction which is unable to support neurofunction eventually leading to neurodegeneration and dementia.

Importantly, our findings have demonstrated that SCF + G-CSF treatment in the chronic phase of severe TBI prevents the TBI-induced microglial pathology in bilateral cortex and hippocampus. SCF + G-CSF treatment shows reduced microglial volume in the ipsilateral hippocampus, increased process complexity of microglia in bilateral hippocampus and cortex, and increased number of microglia in the contralateral hippocampus and cortex, supporting that SCF + G-CSF treatment ameliorates the severe TBI-induced microglial pathology in the chronic phase. In the APP/PS1 mouse model of Alzheimer’s disease, we have observed that SCF + G-CSF treatment also increases the process complexity of microglia and enhances the expression of P2RY12 and TMEM119 positive homeostatic microglia in the brain [80], suggesting that SCF + G-CSF improves the homeostasis of the neuronal microenvironment. The underlying mechanism of SCF + G-CSF-reduced microglial pathology in the chronic phase of severe TBI remains unknown. It has been reported that SCF treatment upregulates the mRNA expression of nerve growth factor, BDNF and ciliary neurotrophic factor, and downregulates the expression of inflammation-associated cytokines, tumor necrosis factor-alpha and interleukin-1beta in cultured microglia [83]. These findings suggest a potential role of SCF in preventing microglial activation and supporting microglial survival.

Microglia play an important role in neural circuit remodeling in both healthy and diseased brains by activity-dependent synaptic pruning [32, 62, 76]. Microglia dynamically interact with synapses and selectively prune nonfunctional synapses [62]. Impaired synaptic pruning may cause synaptic abnormalities and neurobehavioral defects [40]. The findings of the present study reveal that both the excitatory and inhibitory synapses in the hippocampus are extensively generated in the chronic phase of severe TBI, suggesting that the pruning function of microglia is impaired. Pathological changes of microglia in the hippocampus as observed in this study may lead to microglial dysfunction in pruning synapses, which may exacerbate the accumulation of synapses in the hippocampal CA1 in the chronic phase of TBI. Systemic administration of SCF + G-CSF, especially the repeated treatments, in the chronic phase results in enhancing synaptic pruning and preventing the TBI-induced overgrown synapses in the hippocampus. Given that SCF + G-CSF treatment also ameliorates microglial pathology in the hippocampus, the SCF + G-CSF-improved microglial recovery may enhance pruning of the TBI-induced overgrown synapses in the hippocampus. The improved spatial learning/memory by SCF + G-CSF treatment could be associated with the SCF + G-CSF-prevented overgrowth of synapses in the hippocampus in the chronic phase of severe TBI.

Considering the role of SCF + G-CSF on the hematopoietic system, SCF + G-CSF treatment in the chronic phase of severe TBI may also enhance brain repair through bone marrow-derived cells. Our unpublished data have revealed that SCF + G-CSF treatment increases the percentage of monocytes in both the blood and bone marrow and increases the recruitment of bone marrow-derived monocytes/macrophages into the brain during the chronic phase of TBI, and that the SCF + G-CSF-treated monocytes promote neurite outgrowth in vitro. Our findings indicate that SCF + G-CSF treatment may enhance brain repair through multiple mechanisms.

The limitation of this study is that the molecular mechanisms of SCF + G-CSF-enhanced contralateral corticospinal tract sprouting, SCF + G-CSF-ameliorated microglial pathology in the chronic phase of severe TBI, and how SCF + G-CSF treatment enhances microglial function to remove TBI-induced synaptic overgrowth in the chronic phase are not addressed. Further studies are needed to explore the underlying mechanisms of SCF + G-CSF-enhanced brain repair in the chronic phase of severe TBI. In addition, the lack of a sham control group in the in vivo experiment assessing uptake of synapses by microglia (Fig. 8) and the lack of SCF and G-CSF alone treatment in the in vitro experiment confirming SCF + G-CSF-enhanced uptake of synapses by microglia (Fig. 9) are the methodological limitations affecting specificity of the findings.

## Conclusions

Severe TBI in young adult mice causes long-term deficits in neurological function and widespread neuropathological changes in the brain. The neurorestorative efficacy  of SCF + G-CSF-repeated treatments in the chronic phase of severe TBI in improving functional outcome and enhancing brain repair is superior to the SCF + G-CSF single treatment. SCF + G-CSF-repeated treatments initiated at 3 months post-severe TBI (chronic phase) promote contralesional corticospinal tract sprouting into the denervated side of the cervical spinal cord, which is positively correlated with somatosensory-motor function improvement in the affected forelimb/paw. Severe TBI causes microglial dystrophy and microglial loss in the bilateral cortex and contralateral hippocampus and leads to microglial activation in the ipsilateral hippocampus during the chronic phase. In the contralateral cortex, microglial degeneration is associated with the TBI-induced dendritic spine loss. In the hippocampus, microglial pathology is linked to the TBI-induced overgrown synapses. SCF + G-CSF treatment in the chronic phase of severe TBI prevents the TBI-induced microglial loss and dendritic spine loss in the contralateral cortex and re-balances the TBI-induced overgrown synapses in the hippocampus by enhancing microglial function of synaptic pruning. These findings demonstrate the therapeutic potential of SCF + G-CSF treatment in brain repair during the chronic phase of severe TBI.

## Supplementary Information

**Additional file 1: Figure S1.** **Representative images for all experimental groups show the intact corticospinal tract sprouting into the denervated side of cervical spinal cord at segments 6 and 7**. Arrowheads indicate the sprouted intact corticospinal tract fibers that cross the midline of the cervical spinal cord (dashed lines) and extend to the denervated side of the cervical spinal cord. **a** Representative images show the intact corticospinal tract sprouting into the denervated side of cervical spinal cord at segment 6. **b** Representative images show the intact corticospinal tract sprouting into the denervated side of cervical spinal cord at segment 7.

**Additional file 2: Figure S2.** **Representative images for all experimental groups show the intact corticospinal tract (CST) sprouting from non-lesioned side into the denervated side of cervical spinal cord at segments 5 to 7.** The BDA-labeled CST (i.e. the axons are sent from the motor neurons in the contralesional cortex) sprouts into the denervated side of the cervical spinal cord in the chronic phase of severe TBI. Substantial BDA-labeled CST fibers in the denervated side of the cervical spinal cord are seen in the SCF + G-CSF-treated TBI mice. Scale bar: 500 µm.

**Additional file 3: Figure S3.** **SCF + G-CSF treatment promotes neurite outgrowth in vitro**. **a** Representative images show the axon outgrowth in cultured cortical neurons. **b** Quantification data show that SCF-G-CSF treatment promotes neurite outgrowth of cultured cortical neurons. Control: n = 40 neurons, SCF-G-CSF treatment: n = 40 neurons. The data are collected from three independent experiments. Student’s t test. Mean ± SEM. *p < 0.05. (TIF 110 KB)

**Additional file 4: Figure S4.** **SCF + G-CSF-repeated treatments enhance** **the expression of PSD-95 and Gephyrin in the denervated side of cervical spinal cord. a** Representative images of immunofluorescence staining for PSD-95 and Gephyrin in the denervated side of the cervical spinal cord. Maximum intensity Z-projection was used in the images. **b** Quantification data show the relative PSD-95 expression in all experimental groups. **c** Quantification data show  the relative Gephyrin expression in all experimental groups. The blue boxes in panels **b** and **c** indicate the imaging area in the denervated side of cervical spinal cord for data analysis. One-way ANOVA followed by Fisher’s LSD test. Mean ± SEM. *p < 0.05, **p < 0.01. Sham: n = 4, TBI-vehicle: n = 5, TBI-SCF + G-CSF-single treatment: n = 5, TBI-SCF + G-CSF-repeated treatment: n = 5. Scale bar: 10 µm. (TIF 1687 KB)

**Additional file 5: Figure S5.** **PSD-95 and Gephyrin immunopositve puncta in the bilateral cortex are not changed by TBI nor by SCF + G-CSF treatment in the chronic phase of severe TBI**. **a** Representative images show PSD-95 immunopositve puncta in the bilateral cortex. **b** and **c** Quantification data show PSD-95 immunopositve puncta in the contralateral cortex (**b**) and ipsilateral cortex (**c**). **d** Representative images of Gephyrin immunopositve puncta in the bilateral cortex. **e** and **f** Quantification data show Gephyrin immunopositve puncta in the contralateral cortex (**e**) and ipsilateral cortex (**f**). One-way ANOVA followed by Fisher’s LSD test. There are no significant differences among the experimental groups. Sham: n = 3, TBI-vehicle: n = 4, TBI-SCF + G-CSF-single treatment: n = 5, TBI-SCF + G-CSF-repeated treatment: n = 5. Mean ± SEM.

**Additional file 6: Figure S6.** **Neurons in the hippocampal CA1 are not changed in the chronic phase of TBI. a** Representative images of immunofluorescence staining for NeuN positive neurons in the contralateral and ipsilateral hippocampal CA1. **b** Quantification data show  the number of NeuN^+^ cells in the contralateral hippocampal CA1 of all experimental groups. **c** Quantification data show  the number of NeuN^+^ cells in the ipsilateral hippocampal CA1 of all experimental groups. One-way ANOVA. Mean ± SEM. ns: not significant. Sham: n = 3, TBI-vehicle: n = 4, TBI-SCF + G-CSF-single treatment: n = 5, TBI-SCF + G-CSF-repeated treatment: n = 5. Scale bar: 50 µm.

**Additional file 7: Figure S7.** **SCF + G-CSF treatment in the chronic phase of severe TBI reinforces resident microglia to prune synapses in the hippocampal CA1**. **a** A schematic flowchart of the experiment. **b** A schematic diagram shows the imaging regions. **c** Representative confocal images show P2RY12 positive resident microglia that engulf PSD-95 and Gephyrin positive synapses. **d-g** Quantification data show uptake of PSD-95 (**d** and **e**) and Gephyrin (**f** and **g**) positive synapses by the resident microglia in the bilateral hippocampal CA1. Student’s t test. Mean ± SEM. *p < 0.05, ****p < 0.0001. TBI-vehicle: n = 24 microglia (in 4 mice), TBI-SCF + G-CSF treatment: n = 30 microglia (in 5 mice).

**Additional file 8: Figure S8.** **The purity of the primary cultured microglia**. **a** Representative images show Iba1 immunopositive microglia. **b** A pie graph shows the cultured microglia with high purity (97.8%).

**Additional file 9: Figure S9.** **Western blot images taken from four independent experiments. a** Western blot images show the protein expression of PSD-95 and Gephyrin in microglia treated with or without SCF + G-CSF (SG) in three independent experiments. **b** and **c** Western blot images show the protein expression of PSD-95 (**b**) and Gephyrin (**c**) in microglia treated with or without SCF + G-CSF in the fourth independent experiment.

## Data Availability

The datasets generated during the current study are available from the corresponding author on reasonable request.

## Abbreviations

ANOVA: Analysis of variance
BDA: Biotinylated dextran amine
BSA: Bovine serum albumin
CST: Corticospinal tract
FBS: Fetal bovine serum
G-CSF: Granulocyte colony-stimulating factor
Iba1: Ionized calcium binding adaptor molecule 1
MBP: Myelin basic protein
MFI: Median fluorescence intensity
P2RY12: Purinergic Receptor P2Y12
PBS: Phosphate buffered saline
PDL: Poly-d-lysine
PSD-95: Postsynaptic density protein 95
SCF: Stem cell factor
SEM: Standard error of the mean
SYN: Synaptophysin
TBI: Traumatic brain injury
TBS: Tris-buffered saline
TBS-T: TBS containing Tween-20
Tuj1: βIII Tubulin
