# Supplementary figures and images for: SCF + G-CSF treatment in the chronic phase of severe TBI enhances axonal sprouting in the spinal cord and synaptic pruning in the hippocampus
Source: Acta Neuropathol Commun. 2021 Apr 8;9:63. doi: 10.1186/s40478-021-01160-3 (PMC8028149; doi:10.1186/s40478-021-01160-3)

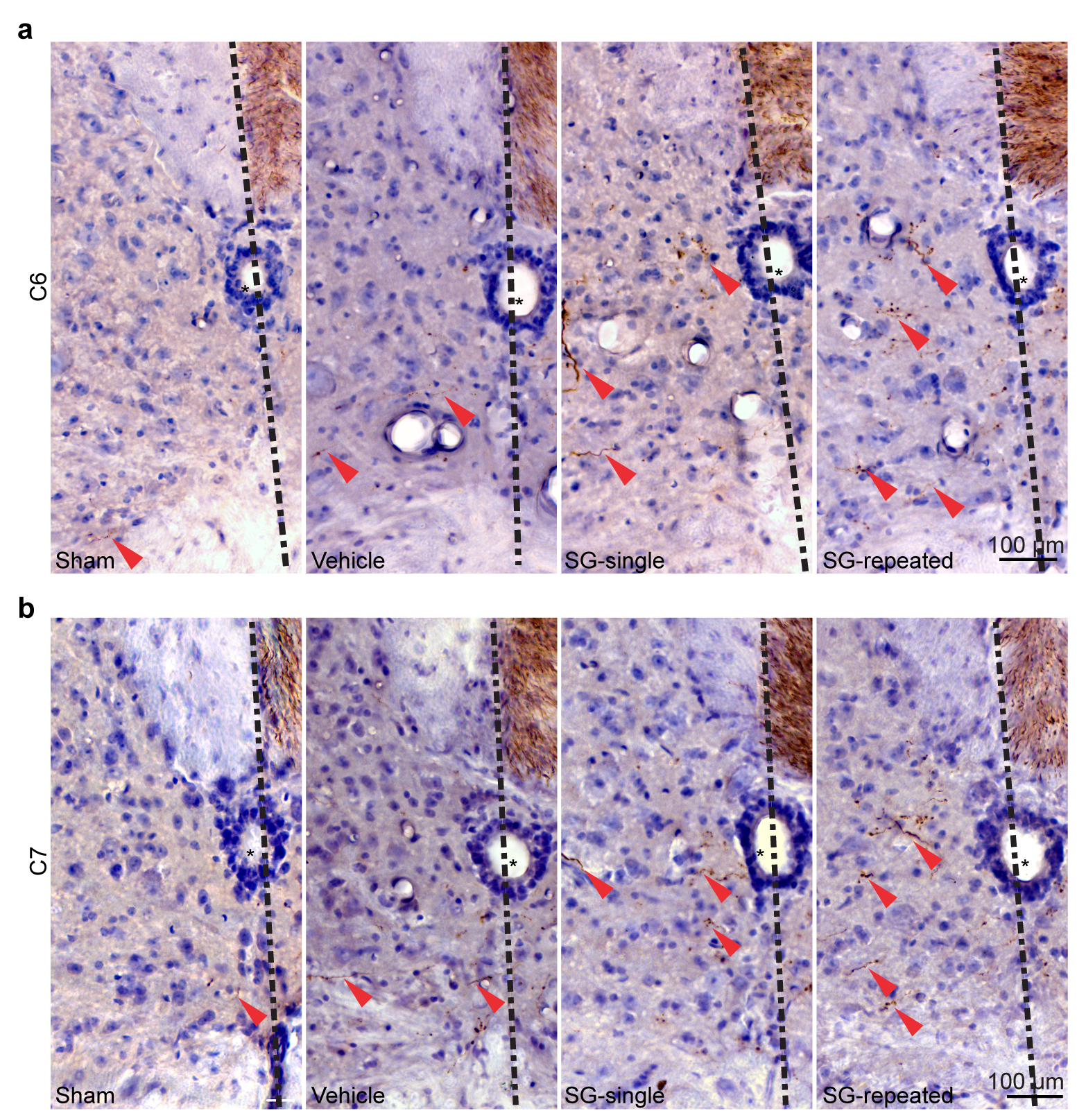

Supplement: Supplementary file 1 — Additional file 1: Figure S1. Representative images for all experimental groups show the intact corticospinal tract sprouting into the denervated side of cervical spinal cord at segments 6 and 7. Arrowheads indicate the sprouted intact corticospinal tract fibers that cross the midline of the cervical spinal cord (dashed lines) and extend to the denervated side of the cervical spinal cord. a Representative images show the intact corticospinal tract sprouting into the denervated side of cervical spinal cord at segment 6. b Representative images show the intact corticospinal tract sprouting into the denervated side of cervical spinal cord at segment 7. [file 40478_2021_1160_MOESM1_ESM.tif]

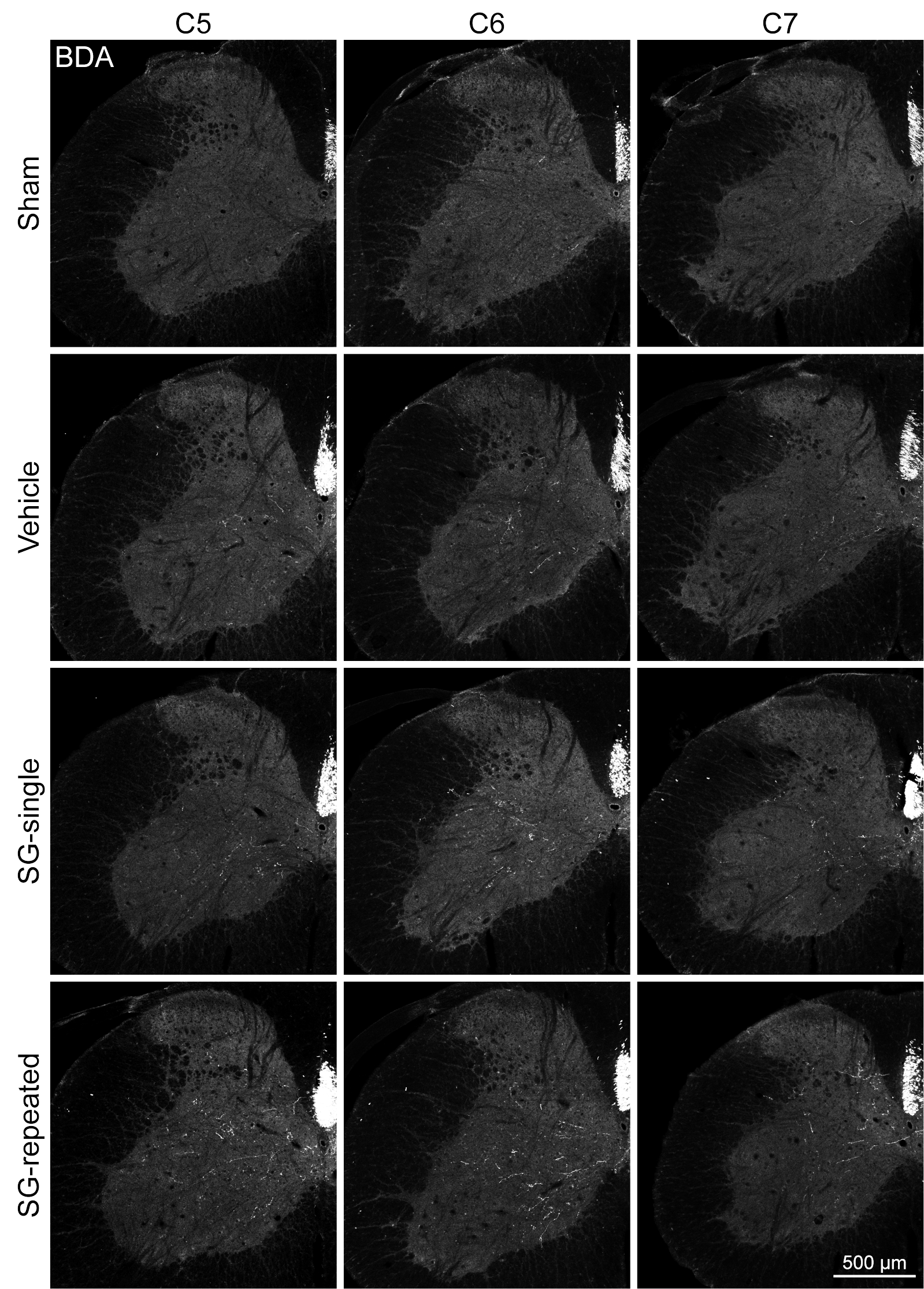

Supplement: Supplementary file 2 — Additional file 2: Figure S2. Representative images for all experimental groups show the intact corticospinal tract (CST) sprouting from non-lesioned side into the denervated side of cervical spinal cord at segments 5 to 7. The BDA-labeled CST (i.e. the axons are sent from the motor neurons in the contralesional cortex) sprouts into the denervated side of the cervical spinal cord in the chronic phase of severe TBI. Substantial BDA-labeled CST fibers in the denervated side of the cervical spinal cord are seen in the SCF + G-CSF-treated TBI mice. Scale bar: 500 µm. [file 40478_2021_1160_MOESM2_ESM.tif]

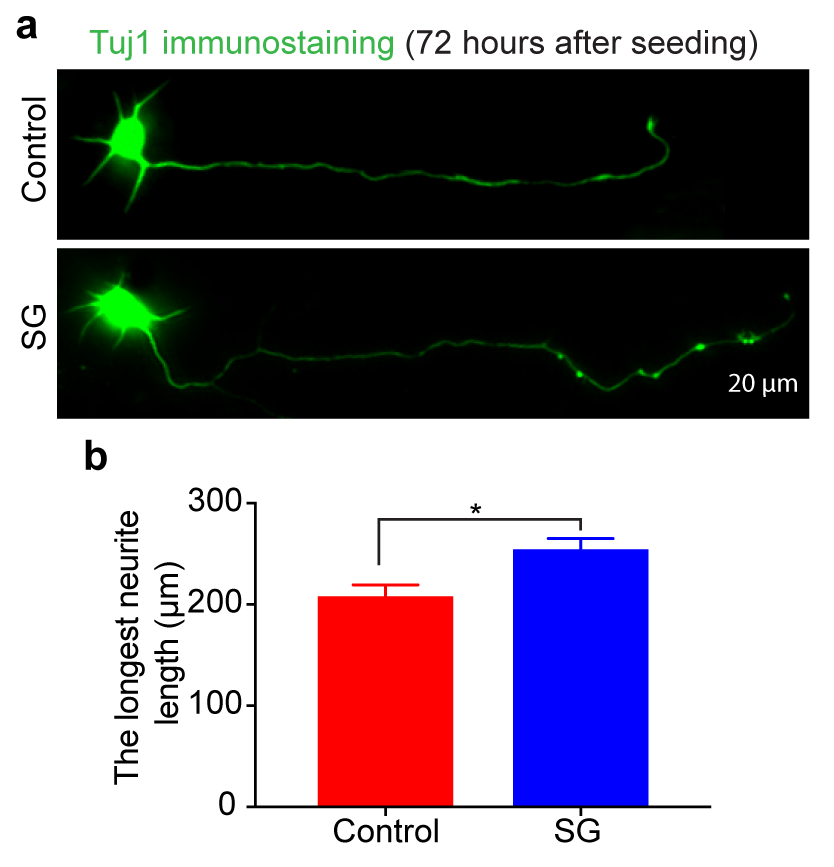

Supplement: Supplementary file 3 — Additional file 3: Figure S3. SCF + G-CSF treatment promotes neurite outgrowth in vitro. a Representative images show the axon outgrowth in cultured cortical neurons. b Quantification data show that SCF-G-CSF treatment promotes neurite outgrowth of cultured cortical neurons. Control: n = 40 neurons, SCF-G-CSF treatment: n = 40 neurons. The data are collected from three independent experiments. Student’s t test. Mean ± SEM. *p < 0.05. (TIF 110 KB) [file 40478_2021_1160_MOESM3_ESM.tif]

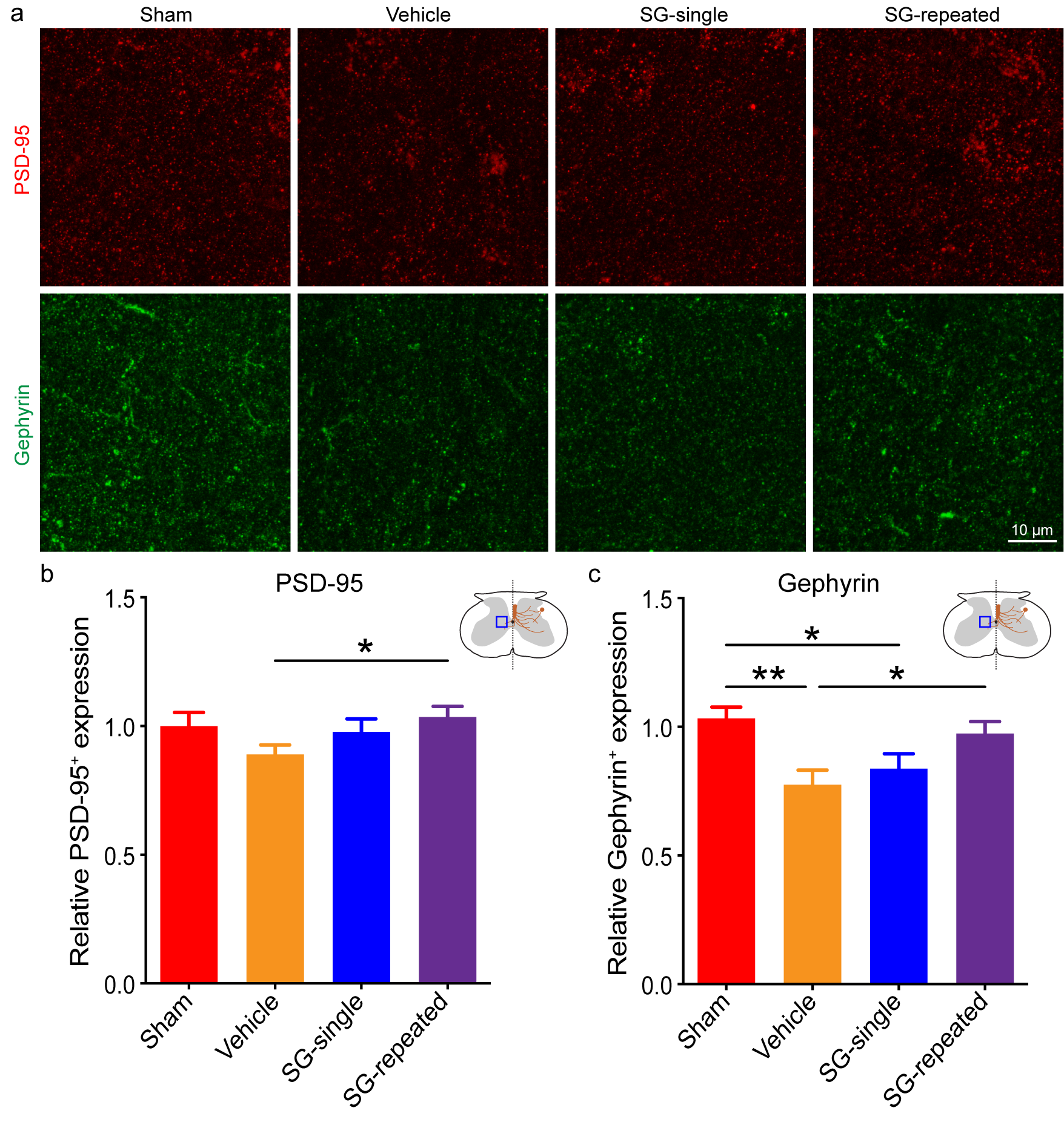

Supplement: Supplementary file 4 — Additional file 4: Figure S4. SCF + G-CSF-repeated treatments enhance the expression of PSD-95 and Gephyrin in the denervated side of cervical spinal cord. a Representative images of immunofluorescence staining for PSD-95 and Gephyrin in the denervated side of the cervical spinal cord. Maximum intensity Z-projection was used in the images. b Quantification data show the relative PSD-95 expression in all experimental groups. c Quantification data show the relative Gephyrin expression in all experimental groups. The blue boxes in panels b and c indicate the imaging area in the denervated side of cervical spinal cord for data analysis. One-way ANOVA followed by Fisher’s LSD test. Mean ± SEM. *p < 0.05, **p < 0.01. Sham: n = 4, TBI-vehicle: n = 5, TBI-SCF + G-CSF-single treatment: n = 5, TBI-SCF + G-CSF-repeated treatment: n = 5. Scale bar: 10 µm. (TIF 1687 KB) [file 40478_2021_1160_MOESM4_ESM.tif]

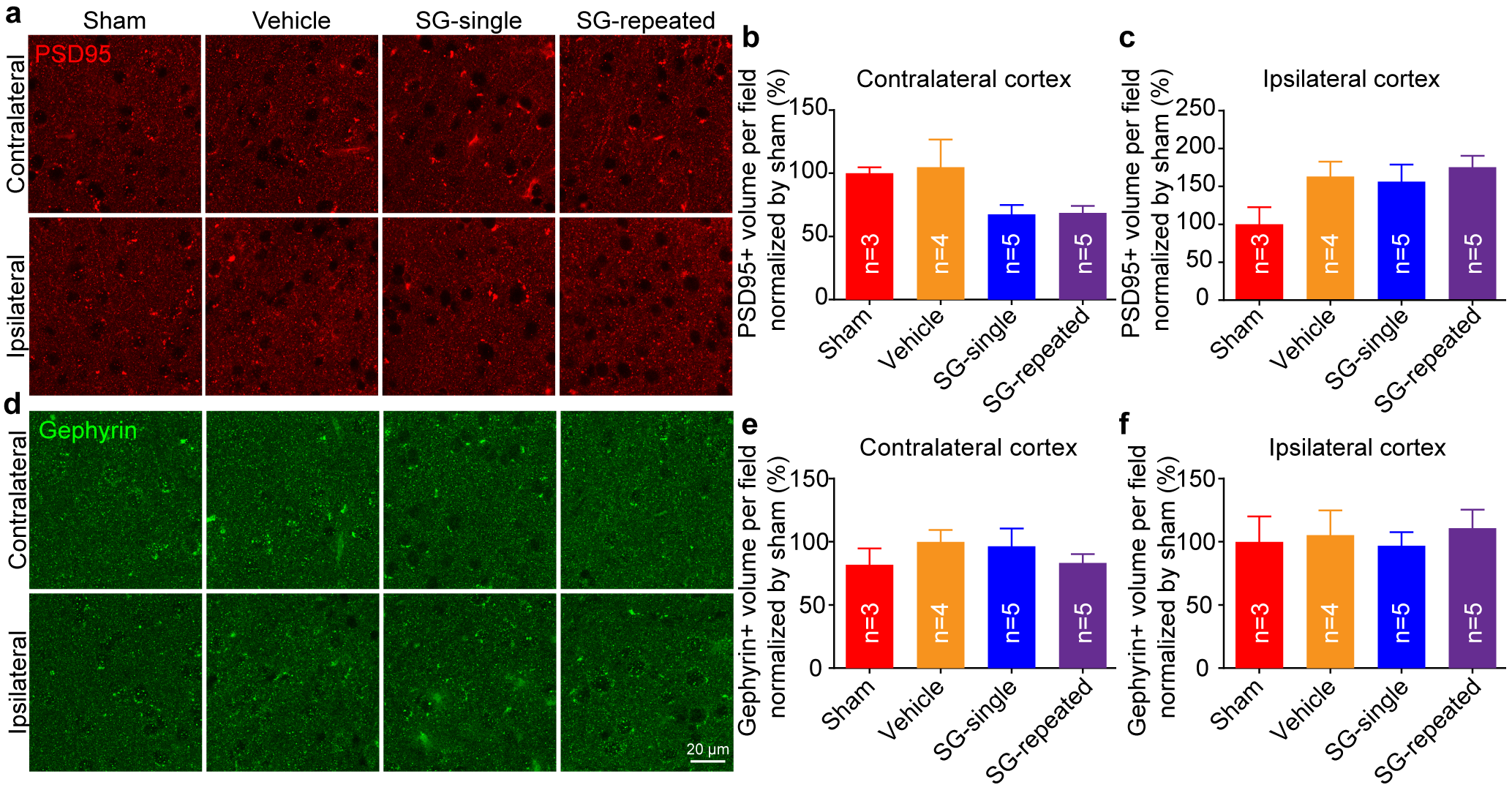

Supplement: Supplementary file 5 — Additional file 5: Figure S5. PSD-95 and Gephyrin immunopositve puncta in the bilateral cortex are not changed by TBI nor by SCF + G-CSF treatment in the chronic phase of severe TBI. a Representative images show PSD-95 immunopositve puncta in the bilateral cortex. b and c Quantification data show PSD-95 immunopositve puncta in the contralateral cortex (b) and ipsilateral cortex (c). d Representative images of Gephyrin immunopositve puncta in the bilateral cortex. e and f Quantification data show Gephyrin immunopositve puncta in the contralateral cortex (e) and ipsilateral cortex (f). One-way ANOVA followed by Fisher’s LSD test. There are no significant differences among the experimental groups. Sham: n = 3, TBI-vehicle: n = 4, TBI-SCF + G-CSF-single treatment: n = 5, TBI-SCF + G-CSF-repeated treatment: n = 5. Mean ± SEM. [file 40478_2021_1160_MOESM5_ESM.tif]

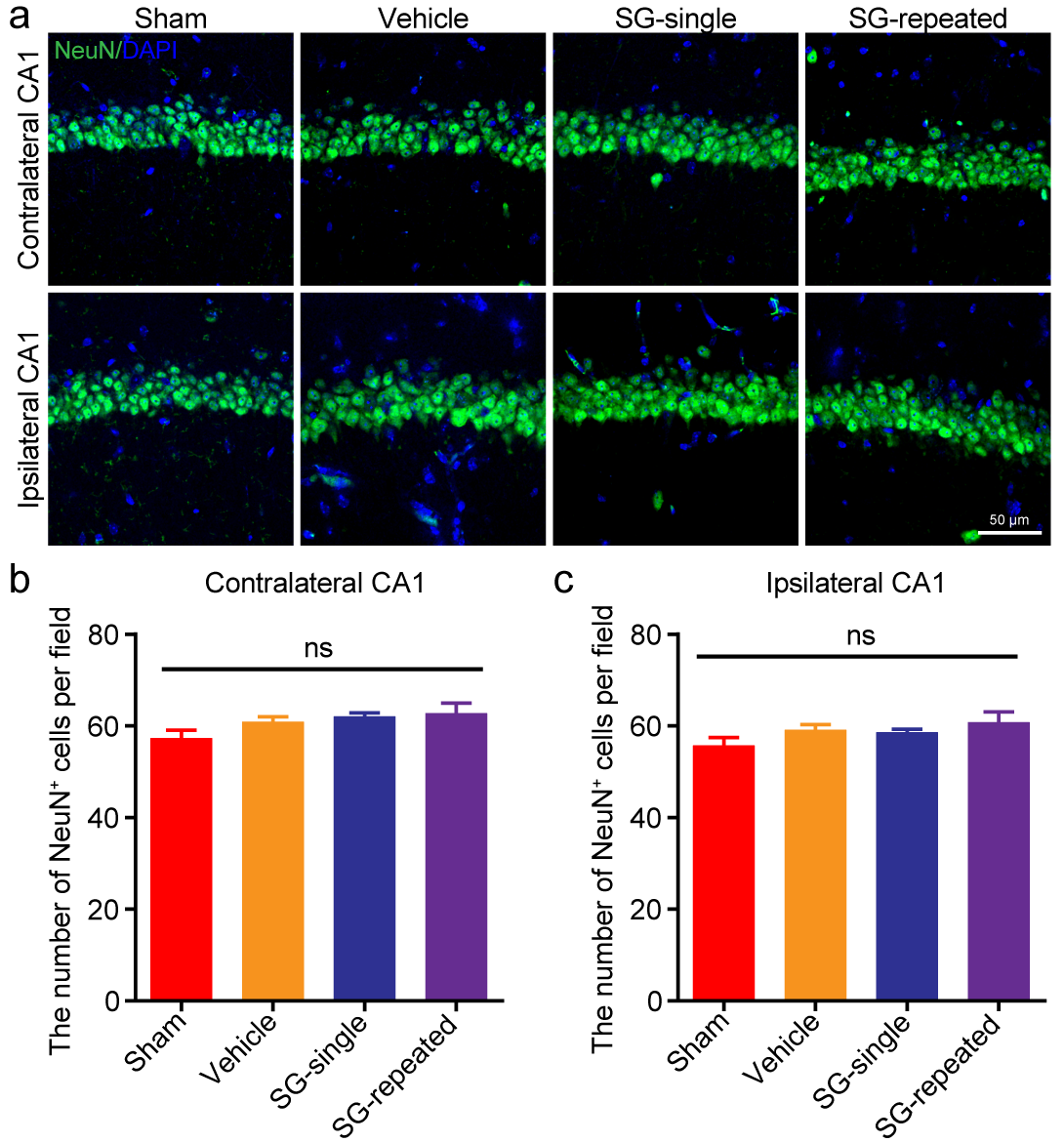

Supplement: Supplementary file 6 — Additional file 6: Figure S6. Neurons in the hippocampal CA1 are not changed in the chronic phase of TBI. a Representative images of immunofluorescence staining for NeuN positive neurons in the contralateral and ipsilateral hippocampal CA1. b Quantification data show the number of NeuN+ cells in the contralateral hippocampal CA1 of all experimental groups. c Quantification data show the number of NeuN+ cells in the ipsilateral hippocampal CA1 of all experimental groups. One-way ANOVA. Mean ± SEM. ns: not significant. Sham: n = 3, TBI-vehicle: n = 4, TBI-SCF + G-CSF-single treatment: n = 5, TBI-SCF + G-CSF-repeated treatment: n = 5. Scale bar: 50 µm. [file 40478_2021_1160_MOESM6_ESM.tif]

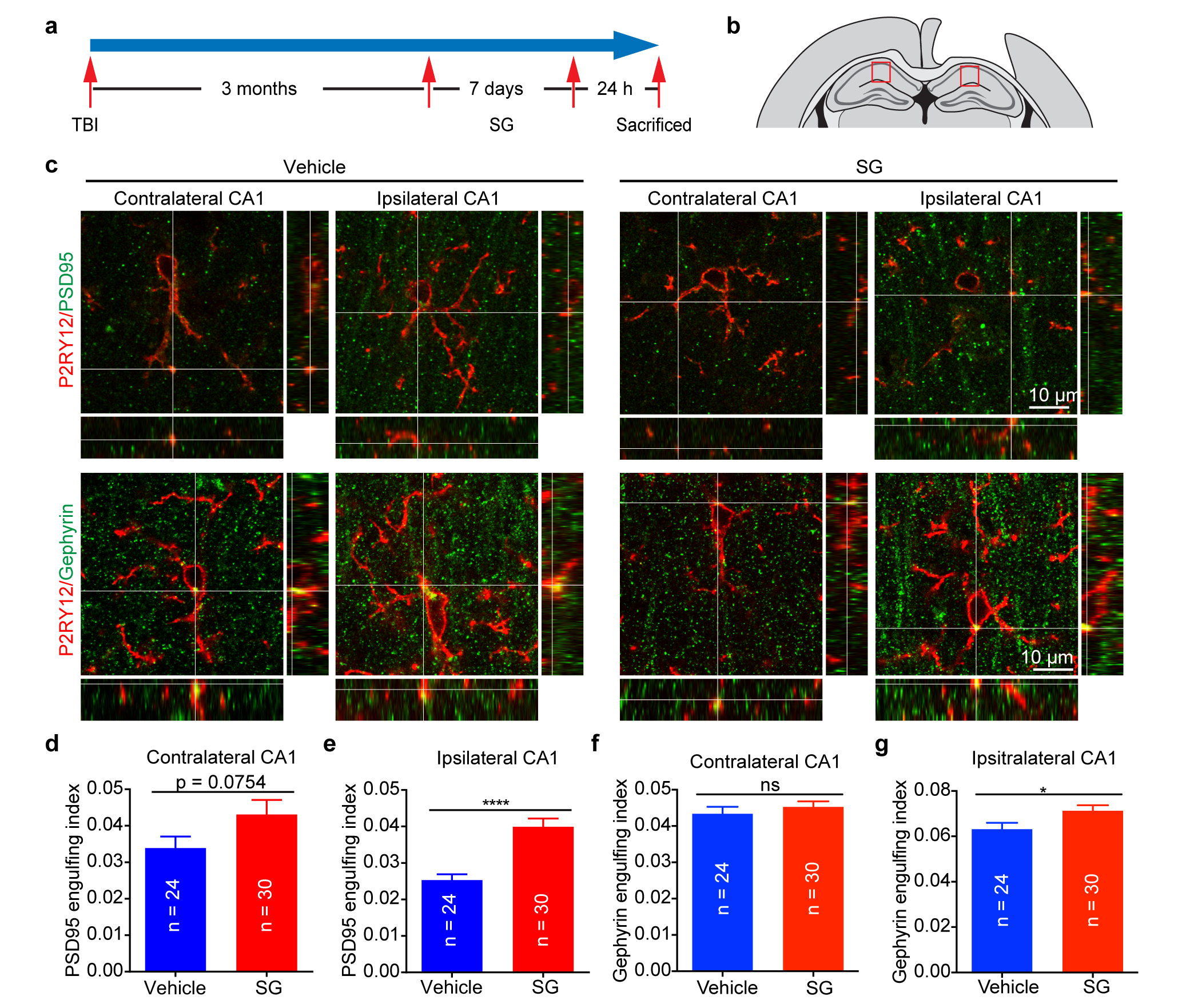

Supplement: Supplementary file 7 — Additional file 7: Figure S7. SCF + G-CSF treatment in the chronic phase of severe TBI reinforces resident microglia to prune synapses in the hippocampal CA1. a A schematic flowchart of the experiment. b A schematic diagram shows the imaging regions. c Representative confocal images show P2RY12 positive resident microglia that engulf PSD-95 and Gephyrin positive synapses. d-g Quantification data show uptake of PSD-95 (d and e) and Gephyrin (f and g) positive synapses by the resident microglia in the bilateral hippocampal CA1. Student’s t test. Mean ± SEM. *p < 0.05, ****p < 0.0001. TBI-vehicle: n = 24 microglia (in 4 mice), TBI-SCF + G-CSF treatment: n = 30 microglia (in 5 mice). [file 40478_2021_1160_MOESM7_ESM.tif]

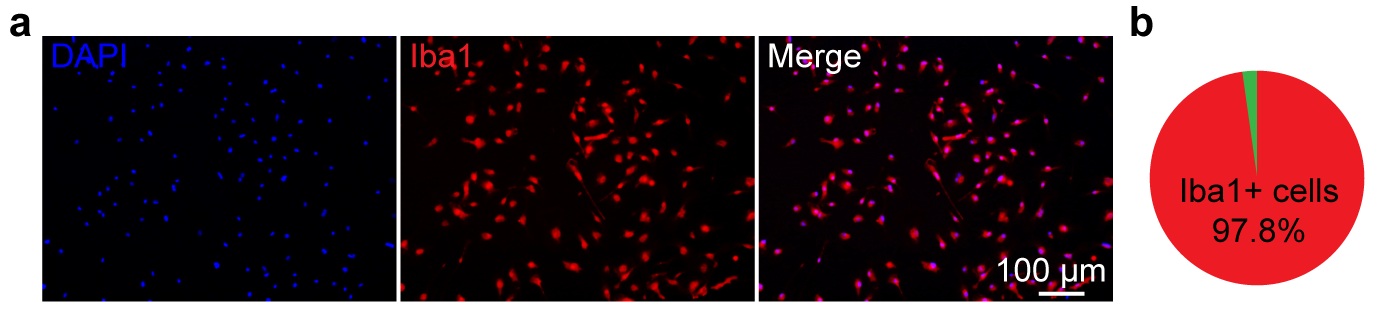

Supplement: Supplementary file 8 — Additional file 8: Figure S8. The purity of the primary cultured microglia. a Representative images show Iba1 immunopositive microglia. b A pie graph shows the cultured microglia with high purity (97.8%). [file 40478_2021_1160_MOESM8_ESM.tif]

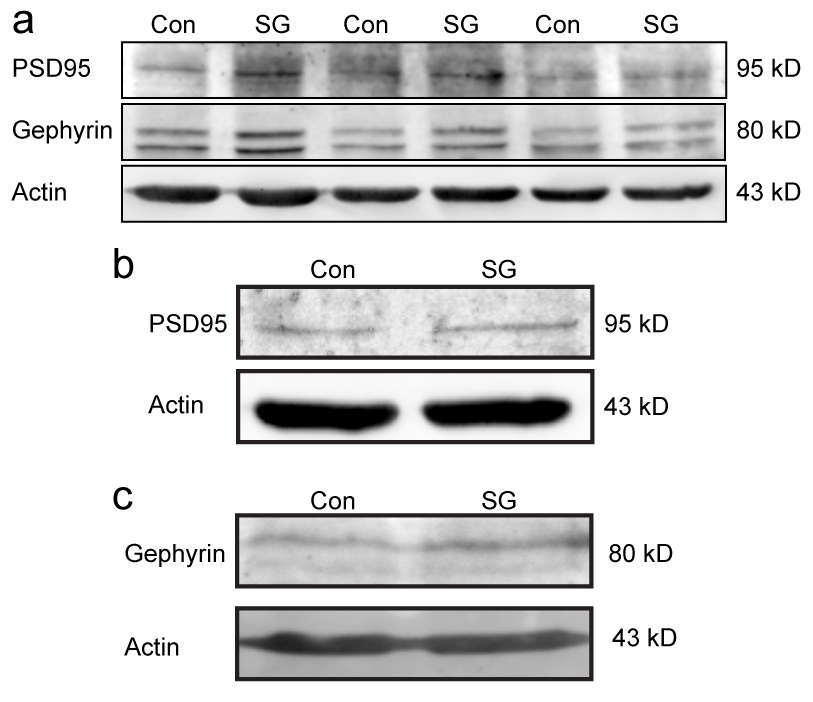

Supplement: Supplementary file 9 — Additional file 9: Figure S9. Western blot images taken from four independent experiments. a Western blot images show the protein expression of PSD-95 and Gephyrin in microglia treated with or without SCF + G-CSF (SG) in three independent experiments. b and c Western blot images show the protein expression of PSD-95 (b) and Gephyrin (c) in microglia treated with or without SCF + G-CSF in the fourth independent experiment. [file 40478_2021_1160_MOESM9_ESM.tif]
